# Supplementary material for: Dense, continuous membrane labeling and expansion microscopy visualization of ultrastructure in tissues
Source: Nat Commun. 2025 Feb 12;16:1579. doi: 10.1038/s41467-025-56641-z (PMC11821914; doi:10.1038/s41467-025-56641-z)
Supplement: Supplementary file 1 — Supplementary Information [file 41467_2025_56641_MOESM1_ESM.pdf]

Supplementary Information

Dense, continuous membrane labeling and expansion microscopy visualization of ultrastructure in tissues.

Contents

Supplementary Notes 4
- Supplementary Note 1 4
- Supplementary Note 2 5
- Supplementary Note 3 5
- Supplementary Note 4 6
- Supplementary Note 5 6
- Supplementary Note 6 7
- Supplementary Note 7 7
- Supplementary Note 8 8
- Supplementary Note 9 8

Supplementary Figures 9
- Supplementary Figure 1 9
- Supplementary Figure 2 10
- Supplementary Figure 3 11
- Supplementary Figure 4 12
- Supplementary Figure 5 13
- Supplementary Figure 6 14
- Supplementary Figure 7 15
- Supplementary Figure 8 16
- Supplementary Figure 9 17
- Supplementary Figure 10 19
- Supplementary Figure 11 20
- Supplementary Figure 12 21
- Supplementary Figure 13 22
- Supplementary Figure 14 23
- Supplementary Figure 15 24
- Supplementary Figure 16 25
- Supplementary Figure 17 26
- Supplementary Figure 18 28
- Supplementary Figure 19 29

Supplementary Figure 20 30
Supplementary Figure 21 31
Supplementary Figure 22 32
Supplementary Figure 23 33
Supplementary Figure 24 34
Supplementary Figure 25 35
Supplementary Figure 26 37
Supplementary Figure 27 39
Supplementary Tables 40
Supplementary Table 1 40
Supplementary Table 2 42
Supplementary Table 3 44
Supplementary Table 4 47
Supplementary Methods 48
Electron microscope imaging, visualization, and analysis 48
Cell preparation 49
Transduction of cells via BacMam virus 50
Brain tissue preparation for mExM 50
mExM for cells 51
mExM for brain tissue slices 52
Immunohistochemistry-compatible mExM 53
Antibody staining of fluorescent proteins for mExM 55
Expansion factor and degree of isotropy analysis 55
Colocalization analysis for mExM images 56
ExM with fGk5b and fGk5b+pGk5b. 57
umExM with GMA anchoring 57
Biotin-DHPE, BODIPY-lipid, mCling staining and proExM 57
IMAXT Consortium Members At Time of Work Performed 59
Supplementary References 59

Supplementary Notes

Supplementary Note 1

We used an azide version of pGk5b (which we named pGk5a) to conjugate gold nanoparticle-DBCO for EM imaging, instead of using pGk5b with gold nanoparticle-streptavidin. We chose this approach because applying streptavidin to the tissue sample typically requires detergent to remove membranes, which could impact downstream processing. We incubated mouse brain tissue sections with 100 µM of the membrane probe pGk5a (pGk5b, with an azide replacing the biotin). We post-labeled the specimens with gold nanoparticles modified with a dibenzocyclooctyne (DBCO) handle, for EM visualization (see **Supplementary Methods** for details). We then imaged the resulting specimens with EM. We saw similar details of intact membranes, organelles, and synapses (**Supplementary Fig. 1a**), when compared to classical OsO_4 membrane visualization (**Supplementary Fig. 1b**), in EM. Note that EM sample processing, involving PFA+glutaraldehyde fixation followed by osmium staining, has been optimized over decades[1–3], and the clear visualization of organelles is protocol-dependent. Early EM protocols often yielded images where mitochondrial cristae and synaptic vesicles were challenging to identify (see Fig 6,9,10 from ref[4]). Similarly, further optimization of EM processing and membrane probe treatment could lead, in principle, to clear visualization of, for example, vesicles and mitochondrial cristae through our lipid stain and EM imaging. However, since our objective was not to optimize our membrane probe treatment and sample processing for EM imaging, but instead to produce EM-like images with expansion microscopy and confocal imaging, we did not further pursue protocol optimization for EM imaging.

Supplementary Note 2

We evaluated the isotropy of mExM expansion by quantitatively comparing pre-expansion structured illumination microscopy (SIM) images to post-expansion confocal images, of the same sample, and calculating the distortion across the images. We imaged fixed U2OS cells expressing mitochondrial matrix-targeted GFP with SIM (see **Supplementary Methods** for details; **Supplementary Fig. 3c**, without anti-GFP labeling; given that resolving the mitochondrial matrix vs. membrane requires 30 nm resolution[5,6], and a classical ExM (that expands ~4x) offers ~60-70 nm resolution[7,8], matrix-targeted GFP is indistinguishable from mitochondrial membrane in the context of the current experiment. We then performed mExM on, and imaged, the very same cells with a confocal microscope. Comparing pre-expansion SIM images of mitochondrial matrix-targeted GFP to post-expansion images of either GFP (with anti-GFP labeling for boosting GFP signals), or pGk5b, we observed the same low distortion (a few percent, over ~10 μm) as was found for previous ExM protocols (see **Supplementary Methods** for details; **Supplementary Fig. 3d** and **Supplementary Fig. 3g**). By comparing the distance between two landmarks in pre- vs. post-expansion images (**Supplementary Fig. 4a** and **Supplementary Fig. 4b**) of the same sample, the expansion factor could be calculated; we obtained an expansion factor (~4.4, **Supplementary Fig. 4c**) similar to what was previously reported[7,8].

Supplementary Note 3

We expressed mitochondrial matrix-targeted GFP or endoplasmic reticulum (ER) membrane-targeted GFP in HEK293 cells via BacMam virus. We performed mExM on the cells

(Supplementary **Fig. 5a-f;** see **Supplementary Methods** for details), and then imaged the expanded cells, so that we could quantify the fraction of mitochondrial matrix- and ER membrane-targeted fluorescent protein signal that also exhibited pGk5b signal (see **Supplementary Methods** for details; in summary, a pixel was considered pGk5b-positive if it was brighter than one standard deviation below the pGk5b mean that was measured across the whole images). As a result, we observed that >99% of the mitochondrial matrix-targeted and ER membrane-targeted GFP signals also exhibited pGk5b signals (n=3 separate cells from 1 culture; **Supplementary Fig. 5g**).

Supplementary Note 4

To enable post-expansion antibody staining, we adopted a commonly used ExM softening protocol[9] (i.e., SDS solution at a high temperature) that can reveal previously unseen structures by preserving protein epitopes through the expansion process[9]. This protocol builds upon post-expansion protein-retention ExM (proExM) protocols[8], as well as tissue proteomics protocols for formalin-fixed paraffin-embedded (FFPE) tissues[10,11]. In brief, we heat the sample for half an hour at 100 ºC and for 2 hours at 80 ºC, in a "fixation reversal" (FR) buffer[12] containing 0.5% PEG20000, 100mM DTT, 4% SDS, in 100mM Tris pH8 (see **Supplementary Table 2** for details).

Supplementary Note 5

Using the softening solution (see **Supplementary Note 4**, **Supplementary Table 2**), we performed mExM with antibody staining (see **Supplementary Methods** for details), using

antibodies against organelle-specific membrane-localized proteins, TOM20 for mitochondria (**Supplementary Fig. 7a**), and Nup98 for the nuclear pore complex (**Supplementary Fig. 7b**). We also labeled myelin using an antibody against myelin basic protein (MBP) (**Supplementary Fig. 7c**). We then quantified the fraction of signals from antibodies against membrane-localized proteins that also contained pGk5b signals, as we did for membrane-targeted GFP in cultured cells (see **Supplementary Note 3**). Since different antibodies may not react to the same sites on the same target protein[9], we used multiple antibodies against the same protein for TOM20 (two separate vendors) and MBP (three separate vendors) to further validate our technology.

Supplementary Note 6

The diameter of axons is known to be diverse across brain regions[13]. However, our finding aligns with measured axon diameters from EM images of the same brain regions (i.e., cortex and dentate gyrus)[13–16].

Supplementary Note 7

Note that none of these lipid stains were reported for tissue application in the ExM context, except mCling, which, in a study using it in tissue, did not provide much in the way of experimental detail[17]. Given the lack of tissue protocol available for these probes, we utilized the staining protocols that had been established for cultured cells. These protocols were applied to standard 4% PFA-fixed tissue, and we performed the most commonly used form of ExM, proExM[8] (see **Supplementary Methods** for details).

Supplementary Note 8

We imaged a random part of the CA1 region of the mouse hippocampus with a 4x objective at 50ms laser exposure time for samples that were stained with biotin-DHPE, BODIPY FL C12, or mCling (**Supp Fig. 19a-c**). We also performed umExM (pGk13a) for comparison (**Supp Fig. 19d**). Qualitatively, umExM generated the highest contrast image, compared to the others. We then measured the signal-to-background (S/B, where the background was determined as the average across images of empty gel regions) for the images obtained from each sample. We found that the S/B for umExM images was many times higher than those of Biotin-DHPE, BODIPY FL C12, and mCling sample images.

Supplementary Note 9

We imaged cilia in the 3rd ventricle to perform continuity analysis, as we did in **Fig. 3o**. However, except for the sample stained with mCling, we were not able to observe any signals. Furthermore, although mCling was able to visualize cilia to some extent, the signals were not dense enough for membranes to be traced, so continuity analysis was not possible (**Supp Fig. 19f-g**).

Supplementary Figures

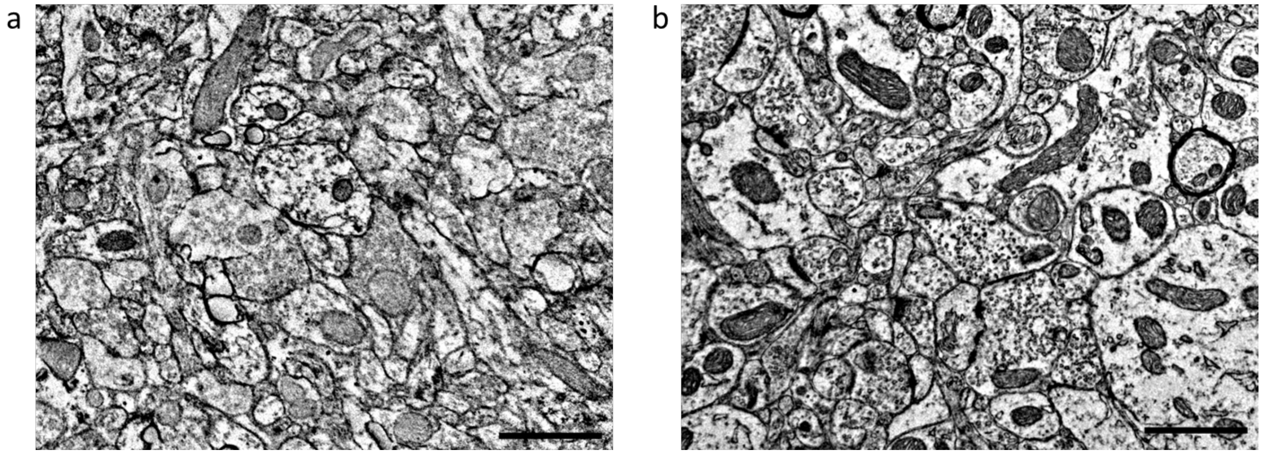

Supplementary Figure 1

Electron microscopy imaging of membrane label (pGk5a)-stained mouse brain slices (hippocampus region). In brief (see **Supplementary Methods** for details), 100 μM of palmitoylated glycine pentalysine peptide, equipped with an azide group (instead of biotin; termed pGk5a), without osmium counterstain (**a**), or no membrane probe but with osmium tetroxide counterstain (**b**), was applied to 100-μm thick tissue slices. Mouse brain tissue was preserved in 4% PFA and 0.1% glutaraldehyde at 4 ºC, and labeled with pGk5a for >16 hours at 4 ºC. The tissue was post-fixed in 2% PFA and 2% glutaraldehyde and labeled with 1.8nm undecagold gold nanoparticles, conjugated to dibenzocyclooctyne to attach to the azide handle on pGk5a. The tissue was counter-labeled with uranyl acetate, embedded in resin, sliced, and imaged on a TEM scope. Since the common practice of uranyl acetate (UA) staining without osmium does not clearly visualize the membrane (Fig. 3 from ref[18], Fig 4. from ref[19]), we reasoned that UA without osmium reacts to proteins as well as the amino groups (i.e., lysines) of pGk5a. We thus decided to use a common UA staining protocol (1% UA for 1 hour at room temperature) to enhance pGk5a signals on top of signals from gold nanoparticles. As a control, the tissue underwent the same protocol as described above, but without membrane probe incubation and with osmium. When labeling membranes with the membrane probe in the absence of the osmium counterstain, the stain appears to label the membranes as with osmium, but with slightly lower contrast. Scale bars: 1 μm.

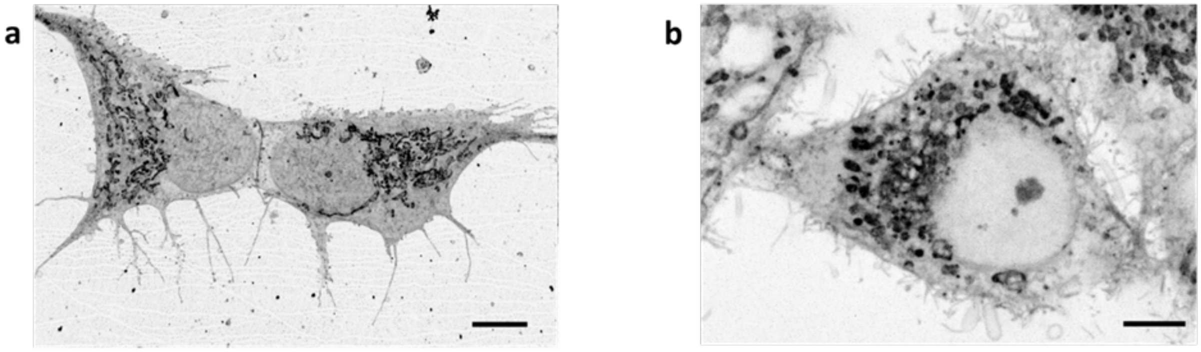

Supplementary Figure 2
(**a**) Representative (n=12 cells from two cultures) single z-plane confocal image of expanded HEK293 cell that underwent the mExM protocol (see **Supplementary Methods**), showing pGk5b staining of the membrane. Image visualized in inverted gray color (dark signals on light background). (**b**) Representative (n=12 cells from two cultures) single z-plane confocal image of expanded HeLa cell that underwent the mExM protocol, showing pGk5b staining of the membrane. Image visualized in inverted gray color. Scale bars: (**a**-**b**) 5 μm.

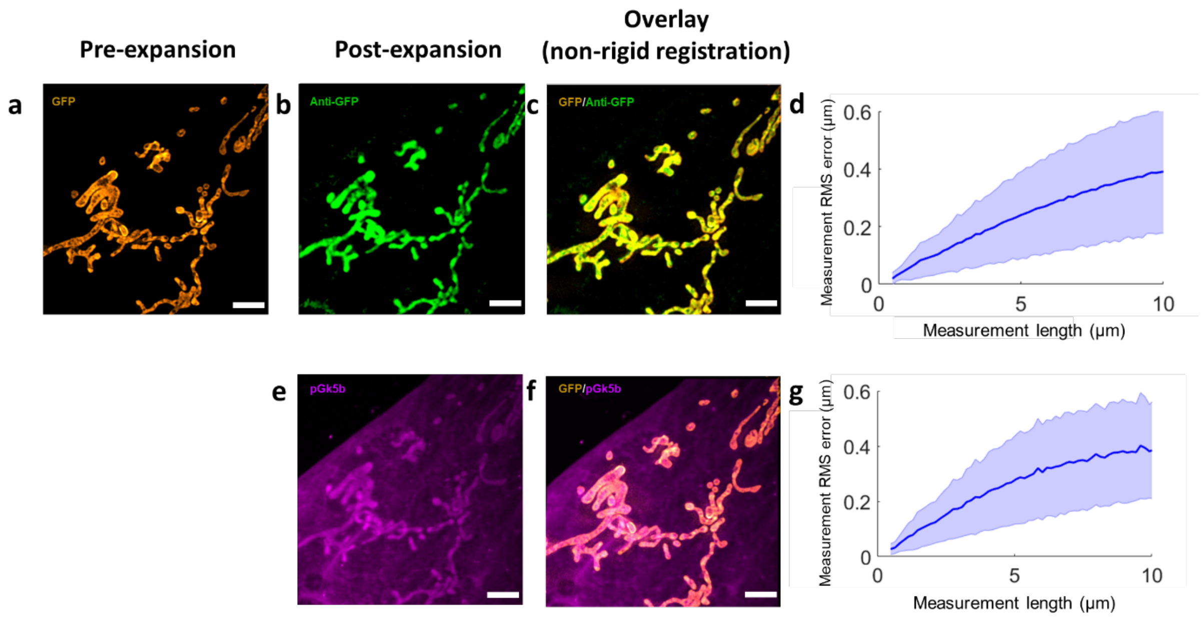

Supplementary Figure 3

(**a**) Representative (n=3 cells from one culture) single z-plane structured illumination microscopy (SIM) image of a pre-expanded U2OS cell expressing mitochondrial matrix-targeted green fluorescent protein (GFP). Image visualized in orange color. (**b**) Single z-plane confocal image of the same U2OS cell as in (**a**), after undergoing the mExM protocol, showing expression of mitochondrial matrix-targeted GFP in the same field of view as shown in (**a**). Image visualized in green color. (**c**) non-rigidly registered and overlaid pre-expansion SIM image of the U2OS cell expressing mitochondrial matrix-targeted GFP in (**a**), and post-expansion confocal image of the same fixed U2OS cell after mExM processing, showing the mitochondrial matrix-targeted GFP channel in (**b**). (**d**) Root mean square (RMS) length measurement error as a function of measurement length, comparing pre-expansion SIM images of fixed U2OS cell expressing mitochondrial matrix-targeted GFP and post-expansion confocal images of the same cells after mExM processing, showing mitochondrial matrix-targeted GFP (blue line, mean; shaded area, standard deviation; n=3 cells from one culture). (**e**) Single z-plane confocal image of the same mExM-expanded fixed U2OS cell as in (**b**), showing pGk5b staining of the membrane in the same field of view as shown in (**a**). Image visualized in magenta color. (**h**) Non-rigidly registered and overlaid pre-expansion SIM image of the U2OS cell expressing mitochondrial matrix-targeted GFP in (**a**) and post-expansion confocal image of the same U2OS cell in (**e**) after mExM processing, showing pGk5b staining. (**g**) Root mean square (RMS) length measurement error as a function of measurement length, comparing pre-expansion SIM images of fixed U2OS cells expressing mitochondrial matrix-targeted GFP, and post-expansion confocal images of the same cell after mExM processing, showing pGk5b staining (blue line, mean; shaded area, standard deviation; n=3 cells from one culture). Scale bars: (**a**,**b**,**c**) 2 µm.

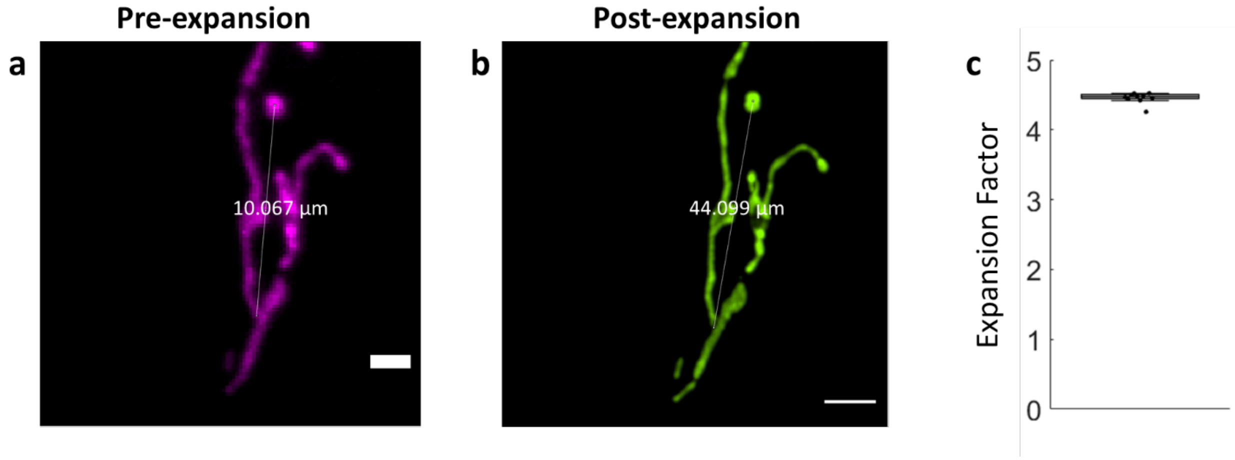

Supplementary Figure 4

(**a-b**) Expansion factor analysis on HEK293 cells, that underwent mExM, after expressing mitochondrial matrix-targeted GFP. We randomly choose two landmark points in pre-expansion images and found the corresponding landmarks in expanded sample images. We then calculated the distance between the points, in both pre- and post-expansion images, and calculated the ratio to obtain the expansion factor. (**a**) Representative (out of 10 cells from two cultures) single z-plane confocal image of pre-expanded HEK293 cell. (**b**) As in (**a**), but post-expansion, for the same field of view shown in (**a**). (**c**) Boxplot showing measured expansion factor (n =10 cells from two cultures; black points, individual measured expansion factor, median, middle line; 1st quartile, lower box boundary; 3rd quartile, upper box boundary; error bars are the 95% confidence interval). Scale bars: (**a**) 2 µm in biological units, (**b**) 10 µm in post-expansion units.

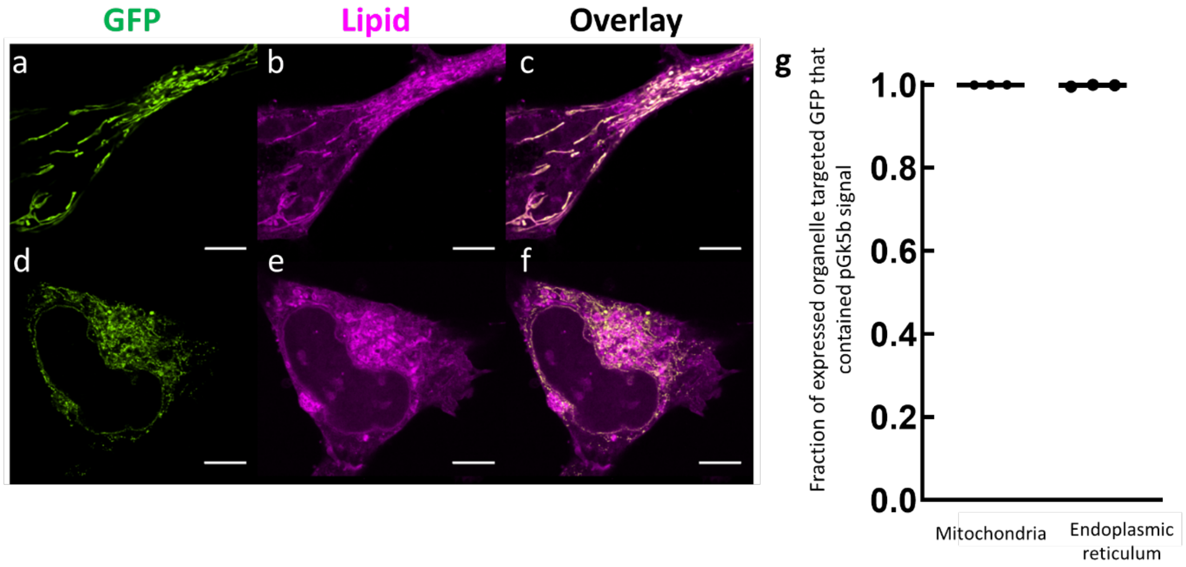

Supplementary Figure 5

(**a**) Representative (n=4 cells from one culture) single z-plane confocal image of expanded HEK293 cell expressing mitochondrial matrix-targeted GFP, after mExM processing. (**b**) Single z-plane confocal image of the same expanded HEK293 cell as in (**a**), showing pGk5b staining in the same field of view as in (**a**). (**c**) Overlay of (**a**) and (**b**). (**d-f**) As in (**a-c**), but for a HEK293 cell expressing ER membrane-targeted GFP (n=3 cells from one culture). (**g**) Fraction of the pixels containing mitochondrial matrix-targeted GFP signal (left, n=3 cells from one culture; black points, individual measured fractions of expressed organelle targeted GFP that contained pGkb5 signal; median, middle line; 1st quartile, lower box boundary; 3rd quartile, upper box boundary; error bars are 95% confidence interval, used throughout unless otherwise noted) or ER membrane-targeted GFP signal (right, n=3 cells from one culture) that also exhibited pGk5b signal, in mExM-processed HEK293 cells (see **Supplementary Methods**). Scale bars are provided in biological units (i.e., physical size divided by expansion factor): (**a-f**) 5 µm.

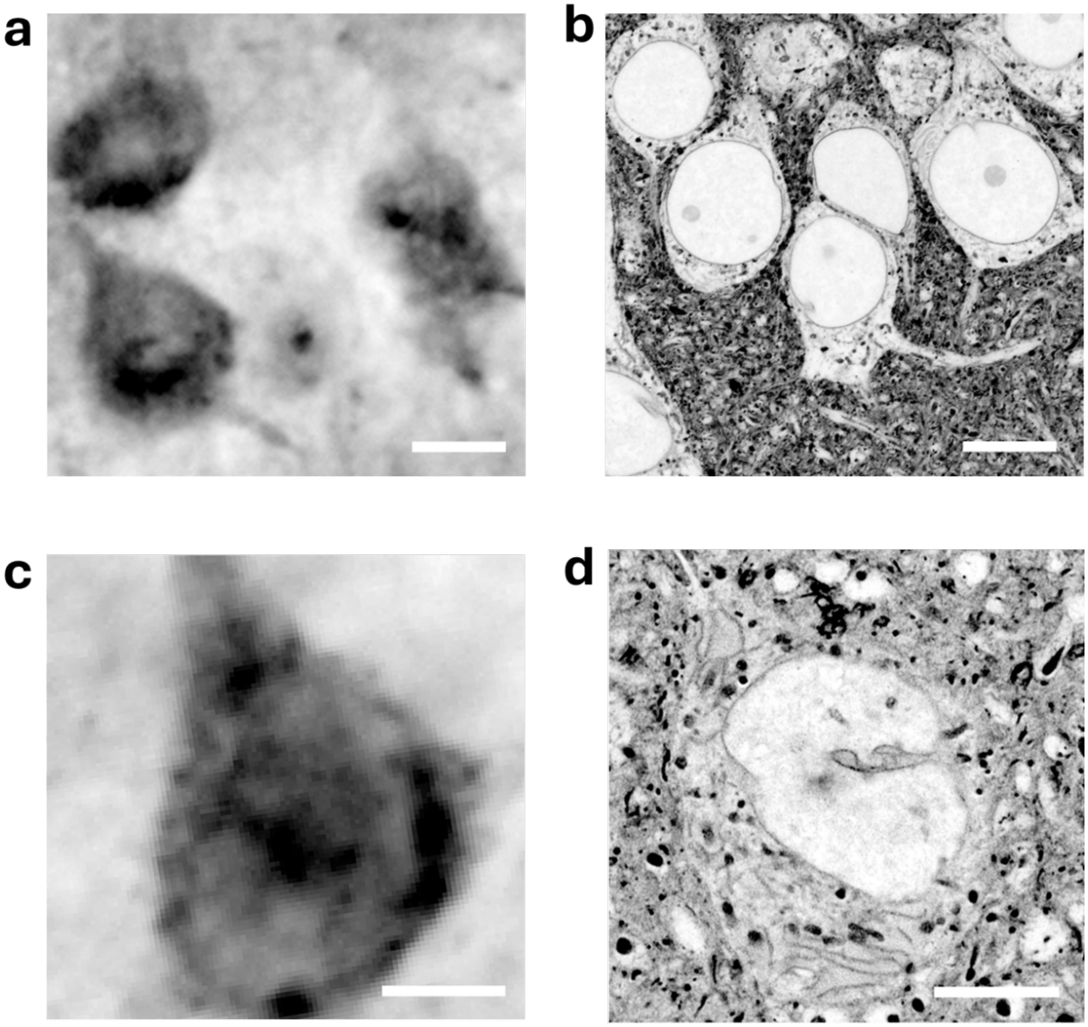

Supplementary Figure 6

(**a**) Representative (n=5 fixed brain slices from two mice) single z-plane confocal image of pre-expanded mouse brain tissue (cortex) showing pGk5b staining. Images visualized in inverted gray color throughout this figure (dark signals on light background). (**b**) Representative (n=5 fixed brain slices from two mice) single z-plane confocal image of expanded mouse brain tissue after mExM processing, showing pGk5b staining. (**c**) As in (**a**) but focused on one cell body. (**d**) as in (**b**) but focused on one cell body. Note that the pre-expansion images of mouse brain tissue in **Supplementary Fig. 6** contain native lipids, which were not removed before imaging. Accordingly, the pre-expansion sample exhibits substantial light scattering and a mismatch in refractive index, which significantly impacts image contrast[20]. Meanwhile, both light scattering as well as mismatch in refractive index are ameliorated by ExM[7]. Scale bars are provided in biological units (i.e., physical size divided by expansion factor): (**a-b**) 10 μm, (**c-d**) 5 μm.

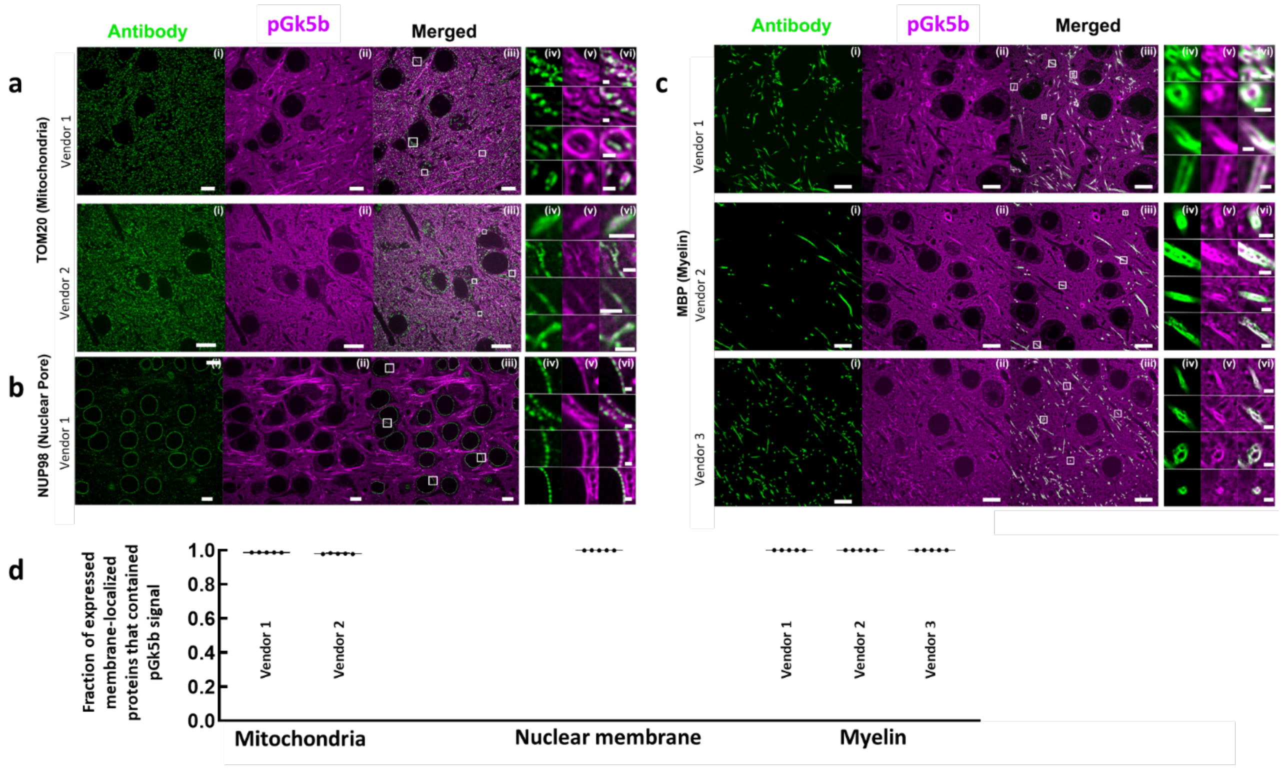

Supplementary Figure 7

(**a.i**) Representative (n=5 cortex or hippocampus regions from the same mouse brain) single z-plane confocal image of expanded mouse brain tissue after mExM processing with immunostaining, showing immunolabeling against the mitochondrial membrane protein Tom20, using antibodies from two separate vendors (see **Supp Table 2** for details). (**a.ii**) Single z-plane confocal image of the specimen of (**a.i**), showing pGk5b staining of the same field of view as in (**a.i**). (**a.iii**) Overlay of (**a.i**) and (**a.ii**). (**a.iv**) Magnified views of white boxed regions in (**a.iii**) showing TOM20 signal (green). (**a.vi**) As in (**a.iv)**, but showing pGk5b signal (magenta). (**a.vii**) Overlay of (**iv**) and (**v**). (**b.**) As in (**a.**), but for the nuclear pore protein NUP98. (**c.**) As in (**a.**), but for myelin basic protein (MBP), using antibodies from three separate vendors (see **Supp Table 2** for details). (**d.**) Fraction of the pixels containing each membrane protein signal, that also contained pGk5b signal, in mExM-processed mouse brain tissue, for each of the antibodies used above (see **Supplementary Methods**; for each box, n=5 cortex or hippocampus regions from the same mouse brain; black points, individual measured fraction of expressed membrane-localized proteins that contained pGk5b signal; median, middle line; 1st quartile, lower box boundary; 3rd quartile, upper box boundary; error bars are the 95% confidence interval). Scale bars: (**i-iii**) 10μm, (**iv-vi**) 1μm.

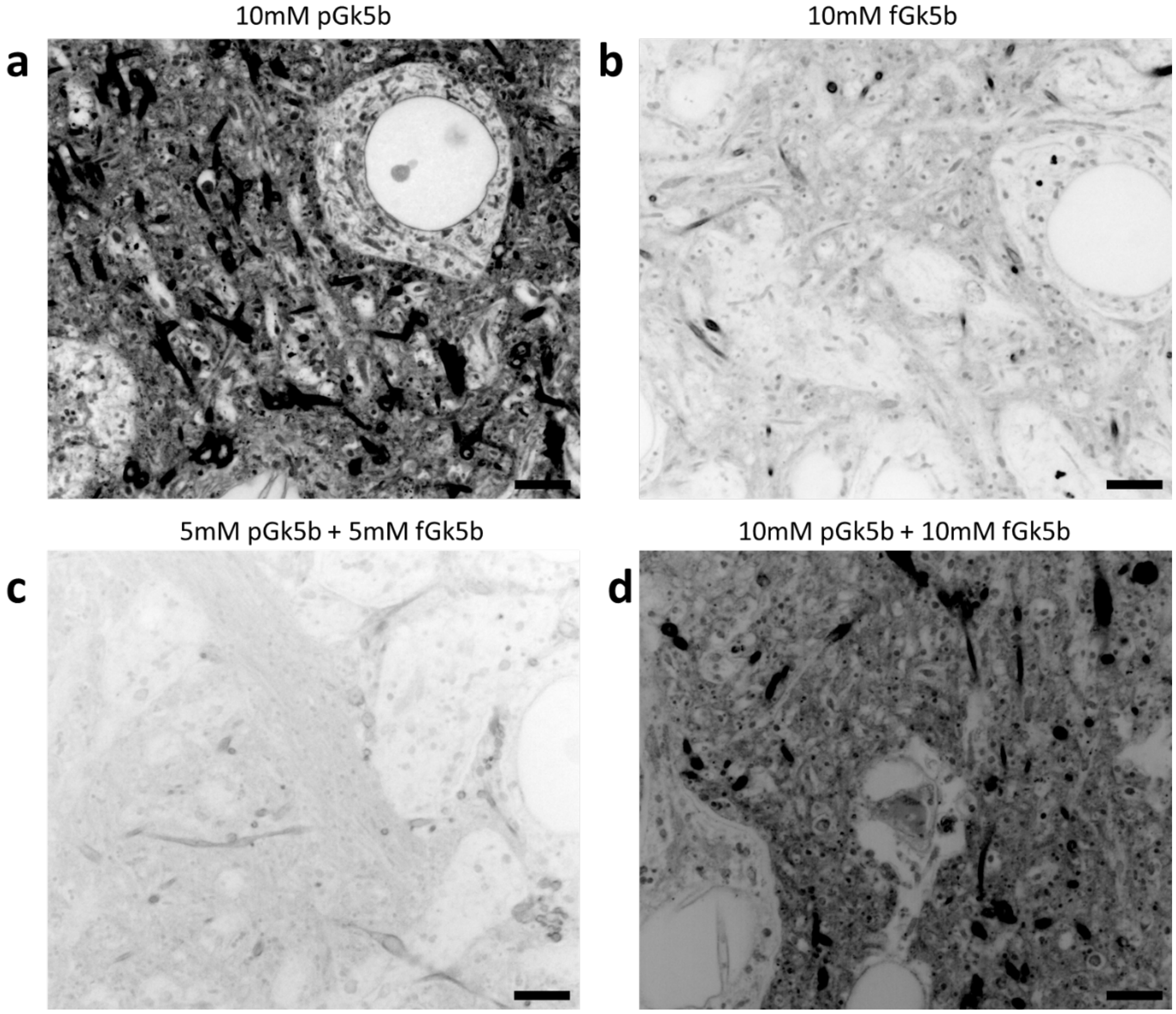

Supplementary Figure 8

We tested two versions of the membrane labeling probe with ExM: (**a**) palmitoyl-glycine-(D-lysine)$_5$-biotin (pGk5b), and (**b**) farnesyl-glycine-(D-lysine)$_5$-biotin (i.e., replacing palmitoyl in pGk5b with farnesyl, fGk5b), as well as (**c-d**) a mixture of pGk5b+fGk5b at varying concentrations. (**a**) Representative (n=5 fixed brain slices from two mice) single z-plane confocal image of expanded mouse brain tissue (hippocampus) after mExM processing, showing pGk5b (i.e., the palmitoylated form of the membrane labeling probe; used at 10mM) staining of the membrane. The image is visualized in inverted gray color. (**b**) as in (**a**), but with a farnesylated form of the membrane labeling probe (used at 10mM, n=5 fixed brain slices from two mice). (**c**) as in (**a**), but with a mixture of 5mM pGk5b + 5mM fGk5b (n=2 fixed brain slices from two mice). (**d**) as in (**a**), but with a mixture of 10mM pGk5b + 10mM fGk5b (n=2 fixed brain slices from two mice). Images (**a**-**d**) are visualized with the same brightness and contrast with ImageJ software. Scale bars are provided in biological units: (**a-d**) 5 µm.

a

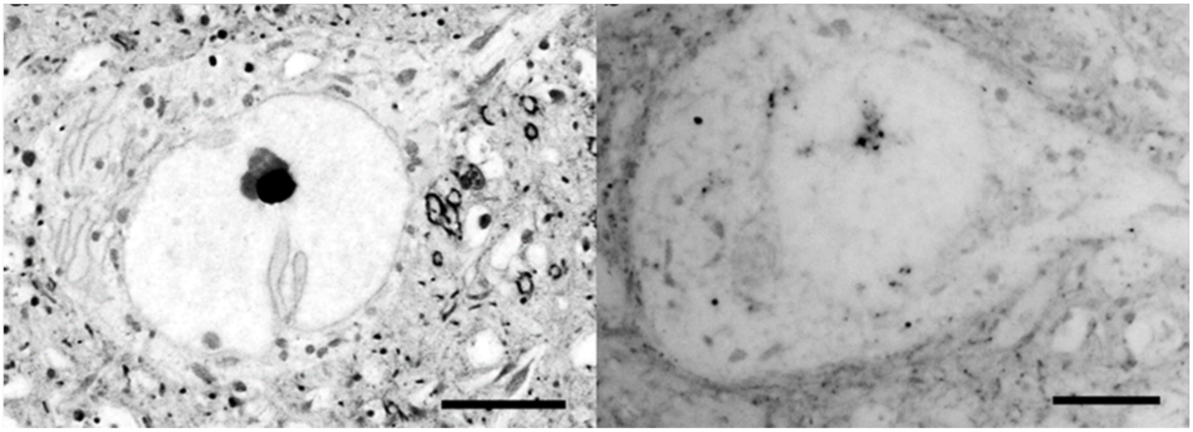

Supplementary Figure 9

(**a**) Effect of the glycine linker attached to the palmitoyl group on the efficacy of membrane labeling in fixed brain tissue. Image visualized in inverted gray color. We tested two versions of the palmitoylated 5 lysine biotin membrane probe: left) one containing a glycine linker attached to the palmitoyl group enabling flexibility of the lipid relative to the peptide carrier, and right) one that does not contain a glycine but in which the lipid is directly attached to the lysine backbone. In the case of the glycine linker, the level of detail we achieve in labeling membranes is superior to that achieved without the glycine linker. Scale bars are provided in biological units (i.e., physical size divided by expansion factor): (**a-b**) 5 μm.

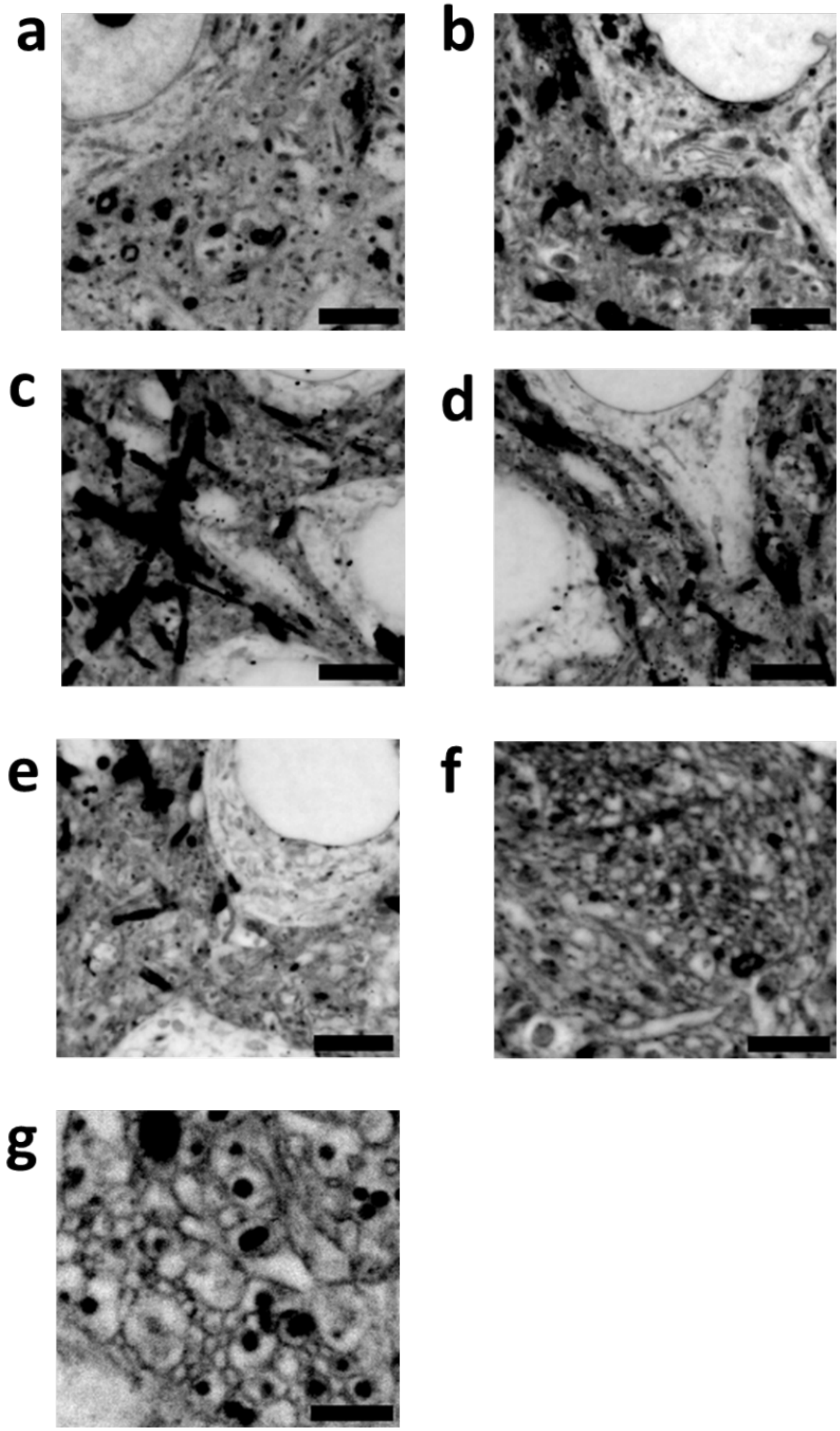
a
b
c
d
e
f
g

Supplementary Figure 10

We tested varying numbers of lysines (i.e., 3, 5, 7, 9, 11, 13, and 19 lysines) in the backbone of the membrane labeling probe while holding other moieties (palmitoyl tail, glycine, and biotin) constant. (**a**) Representative (n=3 fixed brain slices from two mice) single z-plane confocal image of expanded mouse brain tissue (hippocampus) after mExM processing, but with a membrane labeling probe containing 3 lysines, showing the probe staining of the membrane. The image is visualized in inverted gray color. (**b**) - (**g**) As in (**a**) but with membrane labeling probes containing 5, 7, 9, 11, 13, and 19 lysines, respectively. Scale bars are provided in biological units (i.e., physical size divided by expansion factor): (**a-g**) 2 μm.

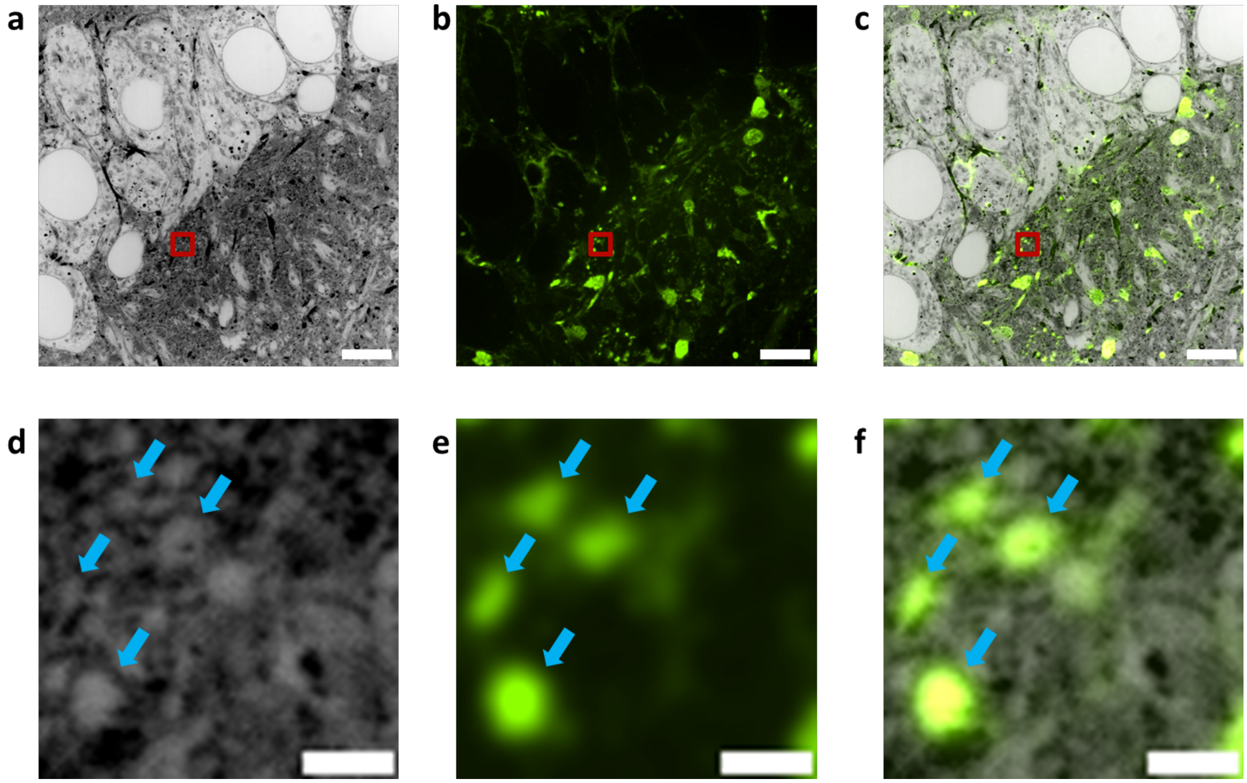

Supplementary Figure 11

(**a**) Representative (n=5 fixed brain slices from two mice) single z-plane confocal image of expanded Thy1-YFP mouse brain tissue (hippocampus) after mExM processing using pGk13b and stained with an anti-GFP antibody to boost the YFP signal, showing the pGk13b staining of the membrane. The image is visualized in inverted gray color. (**b**) Single z-plane confocal image of the specimen of (**a**) showing anti-GFP staining of the same field of view as in (**a**). (**c**) Overlay of (**a**) and (**b**). (**d**) Magnified views of red boxed region in (**a**). (**e**) Magnified views of red boxed region in (**b**). (**f**) Magnified views of red boxed region in (**c**). Scale bars are provided in biological units (i.e., physical size divided by expansion factor): (**a-c**) 5μm, (**d-f**) 0.5μm.

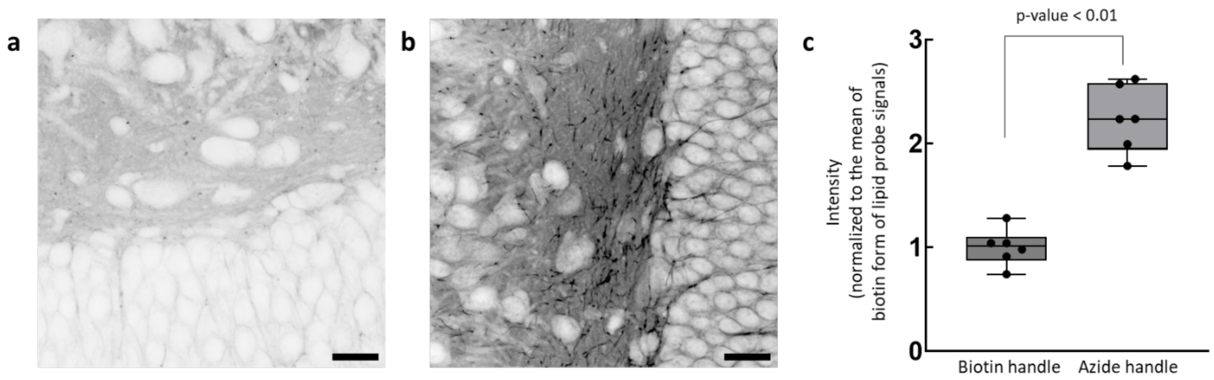

Supplementary Figure 12

The performance of a biotin handle (pGk13b) vs. azide handle (pGk13a) with fixed mouse brain tissues in the context of ExM. Mouse brain tissues were fixed with ice-cold 4% PFA. We applied pGk13b or pGk13a overnight, we then followed the standard ExM protocol[4]. In short, the tissues were processed with AcX, and the ExM gel was formed. After the tissue softening with proteinase K, the probe was fluorescently labeled with fluorescent streptavidin (i.e., cy3-streptavidin, >1 fluorophore per streptavidin), expanded, and imaged (**a**) or with fluorescent DBCO (i.e., cy3-DBCO, 1 fluorophore per DBCO), expanded and imaged (**b**). (**a**) Representative (n = 6 fixed brain slices from two mice) single z-plane confocal image of expanded mouse brain tissue (hippocampus), showing the pGk13b staining of the membrane. The image is visualized in inverted gray color. (**b**) as in (**a**) but with pGk13a, showing the pGk13a staining of the membrane. Images (**a**-**b**) are visualized with the same brightness and contrast with ImageJ software to highlight the difference between the two images. (**c**) The signal intensity of the pGk13b (left; n=6 fixed brain slices from two mice) and the pGk13a (right; n=6 fixed brain slices from two mice). Black points, individual measured average intensity of each image; median, middle line; 1st quartile, lower box boundary; 3rd quartile, upper box boundary; error bars are the 95% confidence interval; p-value, unpaired two-sided t-test between signals from the pGk13b (left) and the pGk13a handle probe (right). Scale bars are provided in biological units (i.e., physical size divided by expansion factor): (**a, b**) 20 μm.

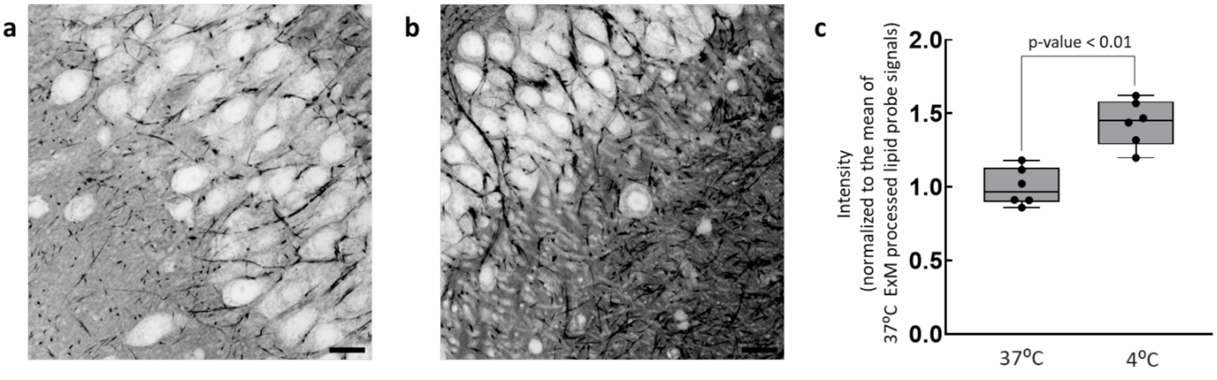

Supplementary Figure 13

pGk13a staining of the membrane of mouse brain tissue fixed with 4%PFA and 0.5% CaCl2 at 4 ºC and proceeded with the standard ExM protocol (37 ºC gelation)[8] vs. modified ExM protocol (i.e., 4 ºC gelation). Mouse brain tissues were fixed with ice-cold 4% PFA and 0.5% CaCl2 fixatives. We applied the pGk13a probe overnight at 4 ºC. We then performed the standard ExM protocol or modified ExM protocol (i.e., 4 ºC gelation instead of 37 ºC gelation). In short, the tissues were processed with AcX, and ExM gel was formed at 37 ºC (**a**) or 4 ºC (**b**). After the tissue softening with proteinase K, the pGk13a was fluorescently labeled with fluorescent DBCO (i.e., cy3-DBCO, 1 fluorophore per DBCO), expanded, and imaged (**a**-**b**). (**a**) Representative (n=6 fixed brain slices from two mice) single z-plane confocal image of expanded mouse brain (hippocampus) tissue showing pGk13a staining of the membrane after the standard ExM protocol. The image is visualized in inverted gray color. (**b**) as in (**a**) but with 4 ºC gelation. Images (**a**-**b**) are visualized with the same brightness and contrast with ImageJ software to highlight the difference between the two images. (**c**) The intensity of 37 ºC gelation (left; n=6 fixed brain slices from 2 mice) and 4 ºC gelation (right; n=6 fixed brain slices from two mice). Black points, individual measured average intensity of each image; median, middle line; 1st quartile, lower box boundary; 3rd quartile, upper box boundary; error bars are the 95% confidence interval; p-value, unpaired two-sided t-test between signals from the 37 ºC gelation (left bar), and 4 ºC gelation (right bar). Scale bars are provided in biological units (i.e., physical size divided by expansion factor): (**a, b**) 20 µm.

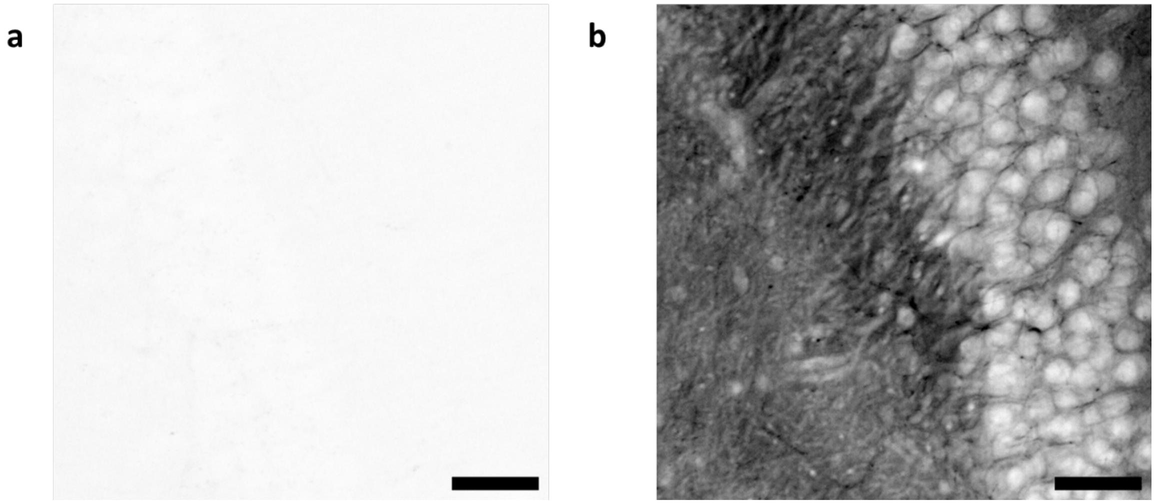

Supplementary Figure 14

(**a**) Representative (n=2 fixed brain slices from two mice) single z-plane confocal image of expanded mouse brain tissue (CA1), that underwent the umExM protocol, but without pGk13a staining of the membrane, showed negligible signals (inverted gray)(**b**) as in (**a**) but with pGk13a staining of the membrane. Images (a-b) were taken under identical optical conditions, and visualized with the same brightness and contrast with ImageJ software. Scale bars are provided in biological units: (**a-b**) 40μm.

a

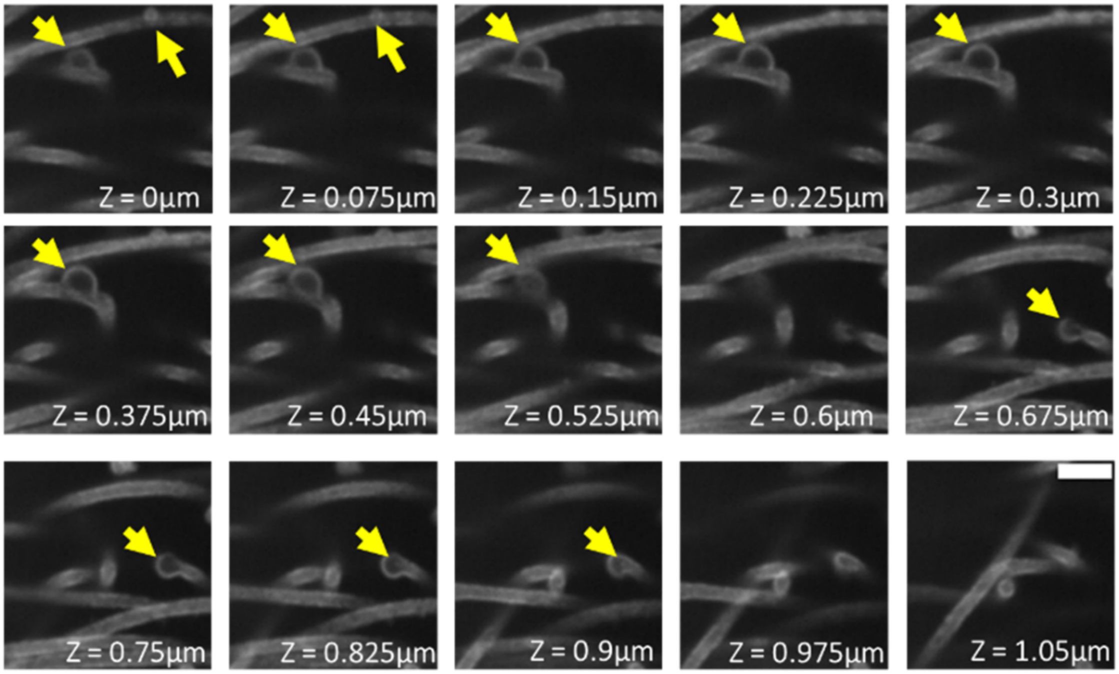

Supplementary Figure 15

(**a**) Fifteen serial sections from the 3D volume rendering in **Fig. 3l, right**. The yellow arrows indicate membrane vesicles. Scale bar in biological units (i.e., physical size divided by expansion factor): 1 μm.

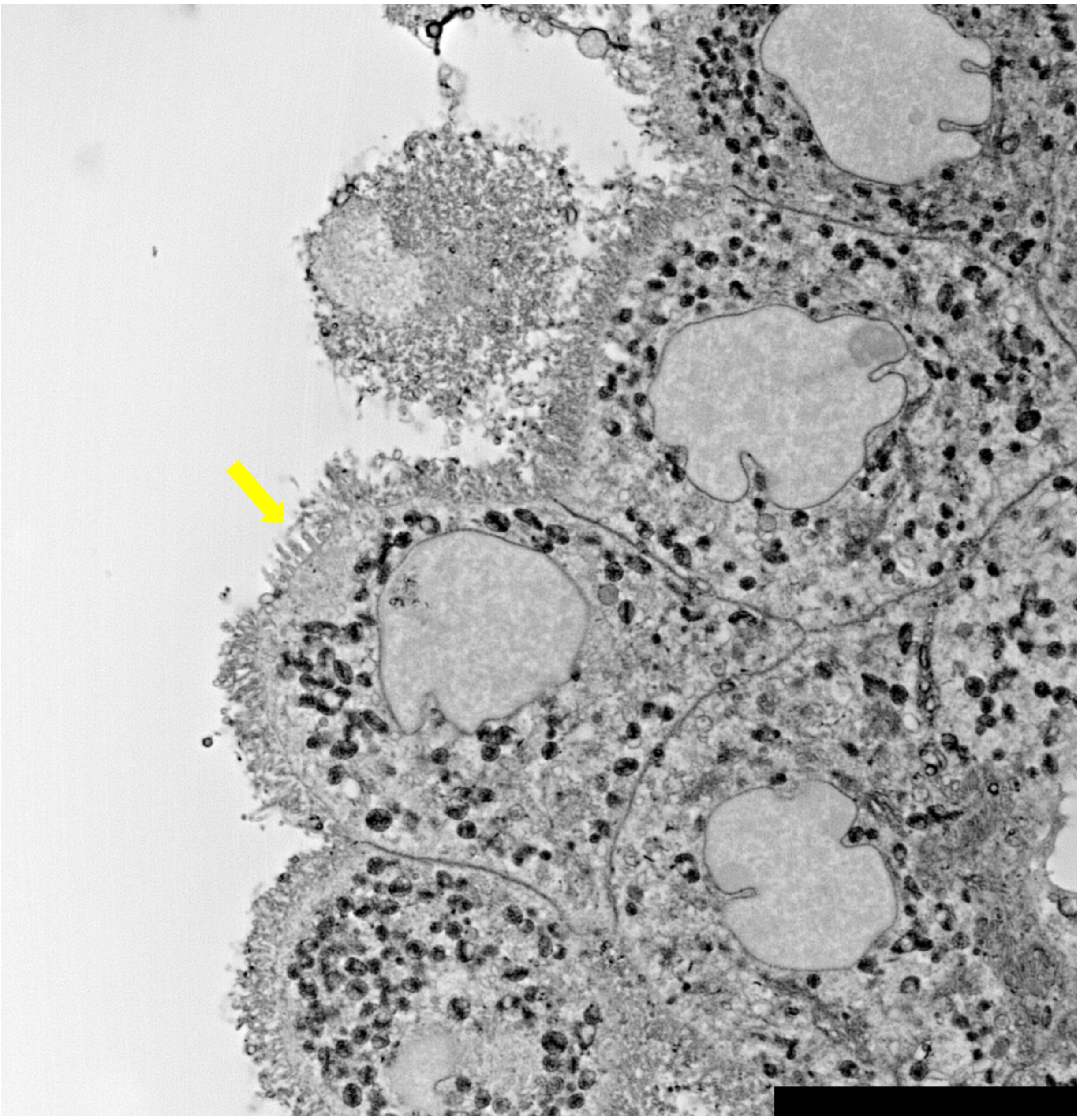

Supplementary Figure 16

Representative (n=5 fixed brain slices from two mice) single z-plane confocal image of expanded mouse brain tissue after umExM processing, showing the pGk13a staining of the membrane in the choroid plexus region. The image is visualized in inverted gray color. Example microvilli in the choroid plexus are pointed at with a yellow arrow. Scale bar in biological units (i.e., physical size divided by expansion factor): 10 μm.

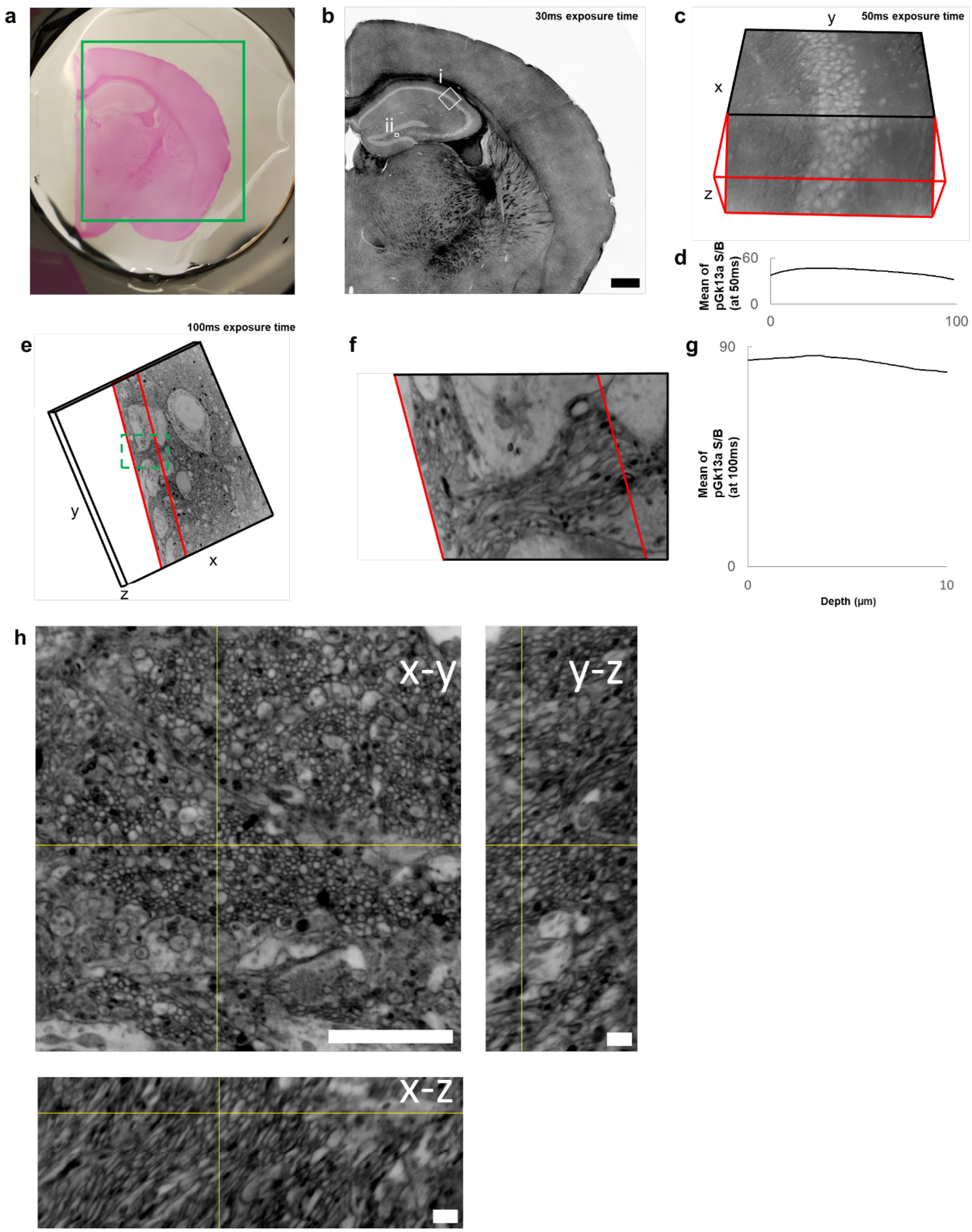

Supplementary Figure 17

(**a**) Photograph of a fixed 100µm thick adult mouse coronal slice that underwent the umExM protocol. (**b**) Single z-plane confocal image of green boxed region in (**a**). Images are taken with a

4x objective at 30ms laser exposure time, and they were stitched with shading correction function via default setting from Nikon Element software version 4.30. pGk13a staining of the membrane visualized in inverted gray color throughout this figure (dark signals on light background). We did not perform any image processing (e.g., denoising or deconvolution) other than stitching for images presented throughout this figure. (**c**) Volume rendering of the white box (i) in (**b**). Images were taken with a 4x objective at 50ms laser exposure time with a z step size of 0.375µm (in biological unit). Unless otherwise noted, clipping planes that are red colored indicate the portion that has been clipped out to expose the inside of the volume for 3D images presented throughout this figure. (**d**) Profile of mean pGk13a signal intensity of XY planes taken along the depth of the volume in (**c**). (**e**) Volume rendering of the white box (ii) in (**b**). Images were taken with a 60x objective at 100ms laser exposure time with a z step size of 0.125µm. (**f**) Magnified view of green boxed region in (**e**). (**g**) Profile of mean pGk13a signal intensity of XY planes taken along the depth of the volume in (**e**). (**h**) Cross-sectional images of dentate gyrus region in 100-µm thick mouse coronal slices that underwent the umExM protocol, showing pGk13a staining of the membrane. Images are taken with a 60x objective at 100ms laser exposure time with a z step size of 0.075µm. Yellow lines indicate the cross-sectional views in y-z and x-z images. Scale bars are provided in biological units (i.e., physical size divided by expansion factor) (**b**) 500µm, (**c**) 340µm (x); 340µm (y); and 100µm (z), (**e**) 62µm (x); 62µm (y); and 20µm (z) (**h**) 5µm (x-y); 1µm (y-z); 1µm (x-z).

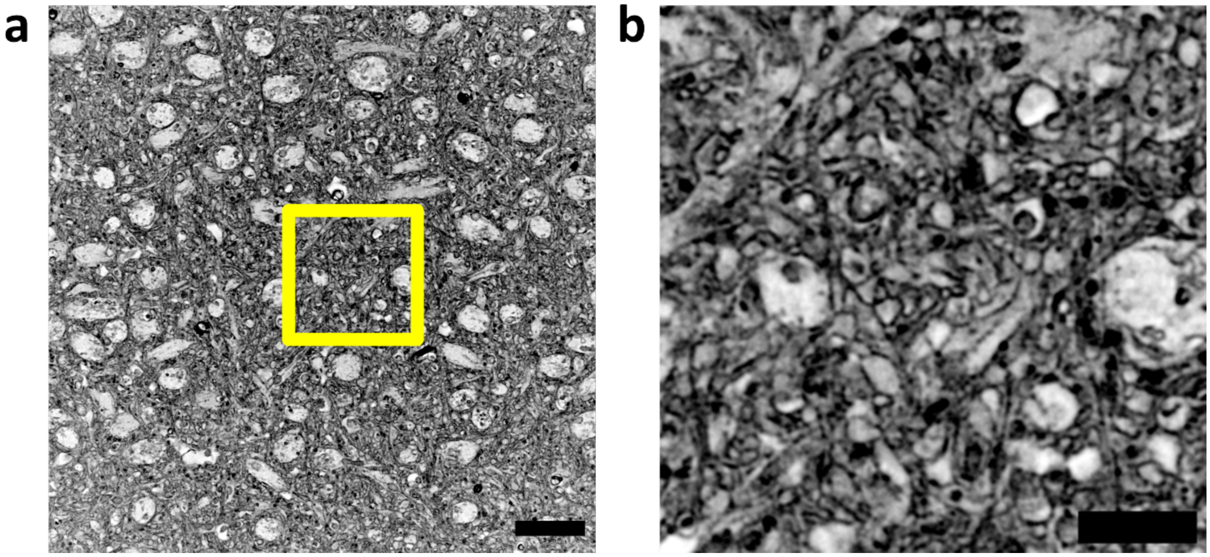

Supplementary Figure 18

(**a**) Representative (n=5 fixed brain slices from two mice) single z-plane confocal image of expanded mouse brain tissue after umExM processing, showing pGk13a staining of the membrane in hippocampal CA2. The image is visualized in inverted gray color. (**b**) Magnified view of yellow boxed region in (**a**). The image is visualized in inverted gray color. Scale bars are provided in biological units (i.e., physical size divided by expansion factor): (**a**) 10 μm, (**b**) 2 μm.

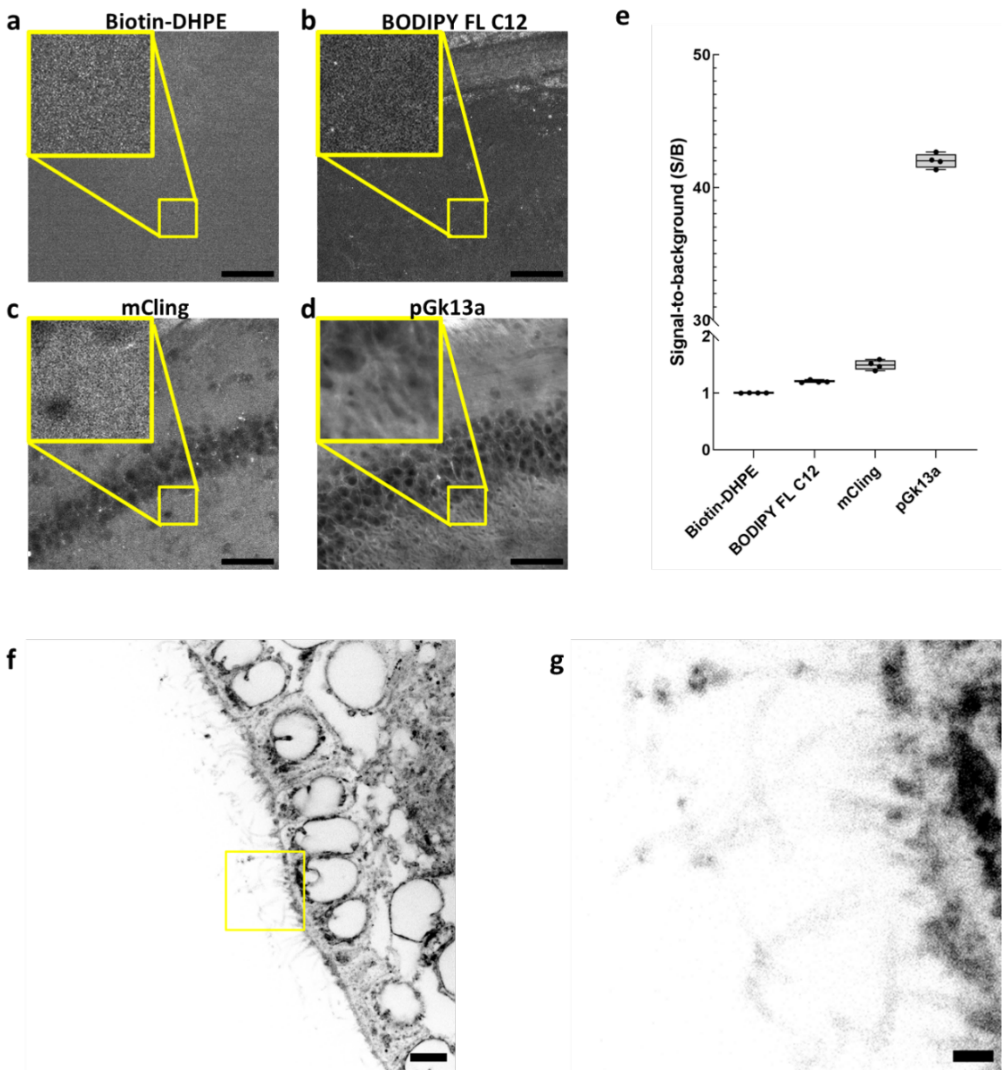

Supplementary Figure 19

(**a**) Representative (n=2 fixed brain slices from two mice) single z-plane confocal image of expanded CA1 region of mouse brain tissue, prepared using the biotin-DHPE staining protocol established in ref[21] and followed by proExM[8], showing biotin-DHPE staining of the membrane. Inset: magnified view of the yellow boxed region. (**b**) as in (**a**) but with the BODIPY staining protocol established in ref[22] and followed by proExM[8], showing BODIPY staining of the membrane. (**c**) as in (**a**), but with the mCling staining protocol established in ref[23] and followed by proExM, showing mCling staining of the membrane. (**d**) as in (**a**), but with the umExM protocol, showing pGk13a staining of the membrane. (**e**) Boxplot showing measured S/B ratio for each of the probes used in **a-d** (n=2 fixed brain slices from two mice; black points, individual measured S/B ratio, median, middle line; 1st quartile, lower box boundary; 3rd quartile, upper box boundary; error bars are the 95% confidence interval). (**f**) as in (**c**), but with imaging of the 3rd ventricle. Image visualized in inverted gray color (dark signals on light background). (**g**) Magnified view of yellow boxed region in (**f**). Scale bars are provided in biological units: (**a-d**) 100 μm, (**f**) 10μm (**g**) 2μm.

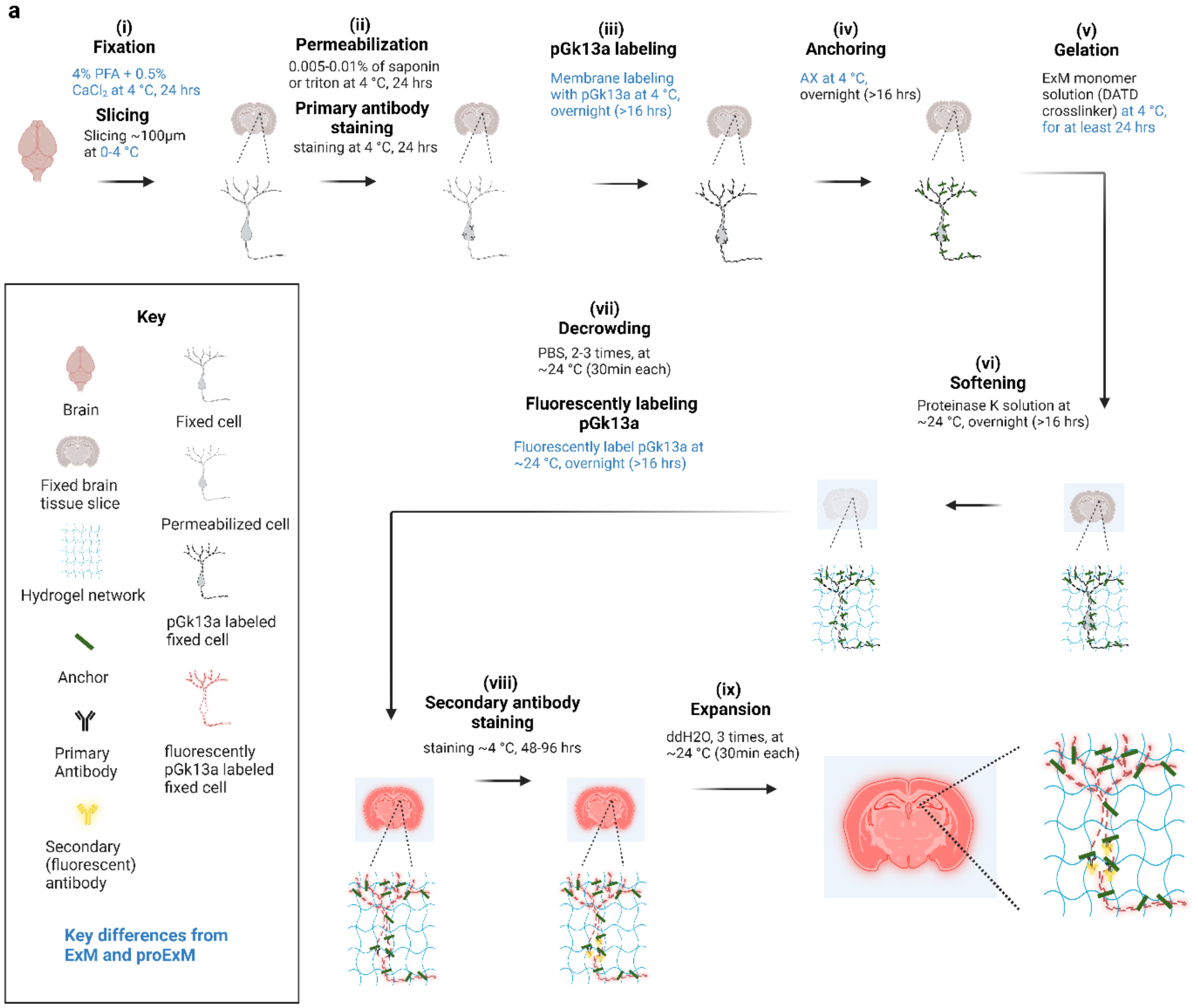

Supplementary Figure 20

(**a**) umExM with pre-expansion antibody staining workflow schematic. Blue-colored captions highlight the key differences from ExM[7] and proExM[8], whereas black captions highlight steps similar to those of earlier protocols. (**a.i**) A specimen is chemically fixed with 4% PFA + 0.5% $CaCl_2$ at 4 ºC for 24 hours. The brain is sliced on a vibratome at 100 μm thickness at 4 ºC. (**a.ii**) The specimen is treated with either 0.005-0.01% detergent (i.e., saponin or triton) at 4 ºC overnight (unless otherwise noted, overnight means >16 hours throughout the figure). Then specimen is incubated with a primary antibody. (**a.iii**) The specimen is treated with pGk13a at 4 ºC overnight. (**a.iv**) The specimen is treated with AX at 4 ºC overnight. (**a.v**) The specimen is embedded in an expandable hydrogel (made with DATD crosslinker[24]) at 4 ºC overnight. (**a.vi**) The sample (specimen-embedded hydrogel) is chemically softened with enzymatic cleavage of proteins (i.e., non-specific cleavage with proteinase K) at room temperature (~24 ºC), overnight. (**a.vii**) Then, the sample is treated with PBS to partially expand it. The pGk13a, that is anchored to the gel matrix, is fluorescently labeled via click-chemistry (i.e., DBCO-fluorophore) at room temperature, overnight. (**a.viii**) Then the sample is incubated with a secondary antibody at 4 ºC for 2-3 days. (**a.ix**) The sample is expanded with water at room temperature for 1.5 hours (exchanging water every 30 minutes).

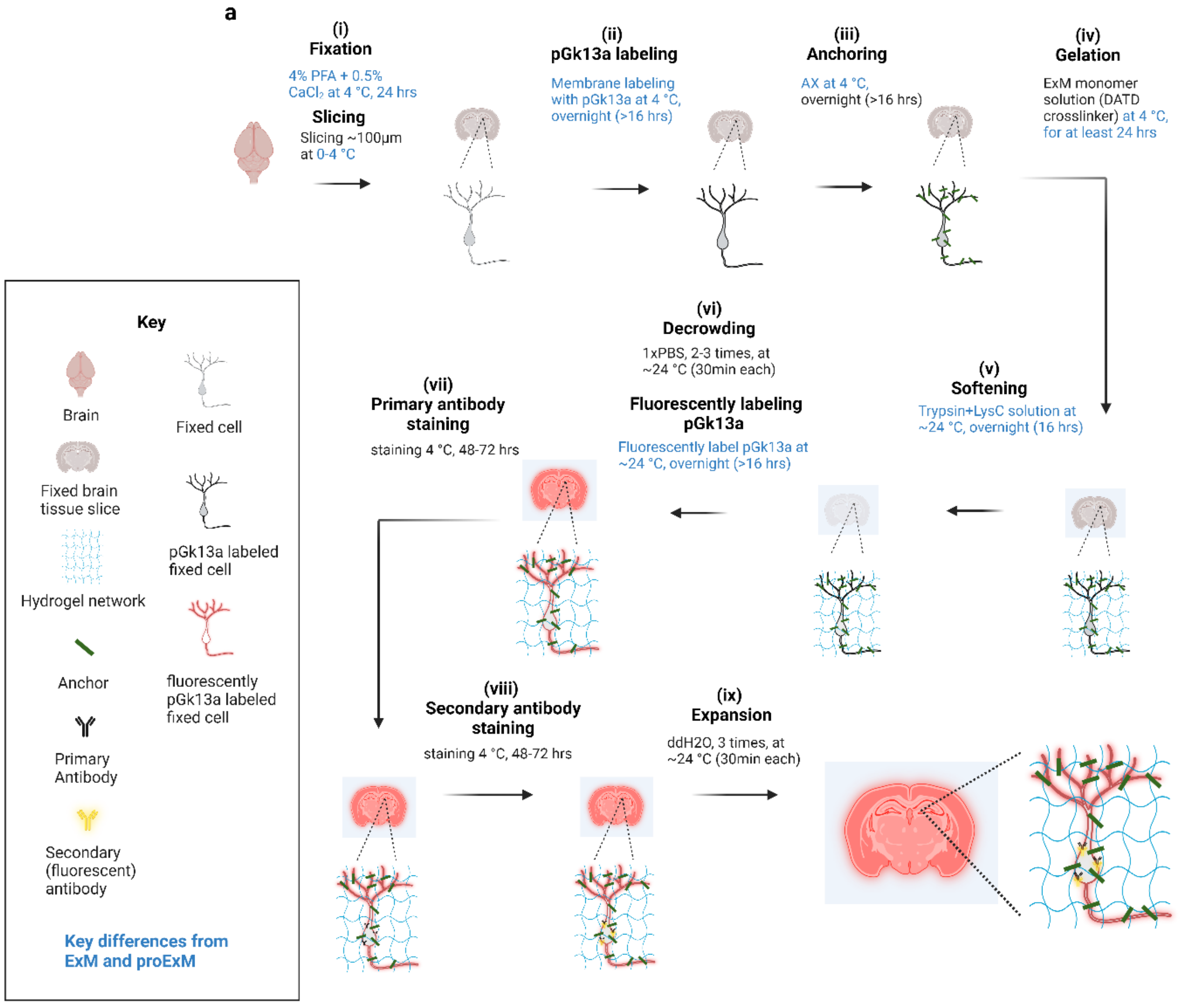

Supplementary Figure 21

(**a**) umExM with post-expansion antibody staining workflow schematic. Blue-colored captions highlight the key differences from ExM[7] and proExM[8], whereas black captions highlight steps similar to those of ExM and proExM. (**a.i**) A specimen is chemically fixed with 4% PFA + 0.5% $CaCl_2$ at 4 ºC for 24 hours. The brain is sliced on a vibratome at 100µm thickness at 4 ºC. (**a.ii**) The specimen is treated with pGk13a at 4 ºC overnight (unless otherwise noted, overnight means >16 hours throughout the figure). (**a.iii**) The specimen is treated with AX at 4 ºC overnight. (**a.iv**) The specimen is embedded in an expandable hydrogel (made with DATD crosslinker[24]) at 4 ºC overnight. (**a.v**) The sample (specimen-embedded hydrogel) is mechanically softened with enzymatic cleavage of proteins (i.e., specific cleavage with Trypsin and LysC) at room temperature (~24 ºC), overnight. (**a.vi**) Then, the sample is treated with PBS to partially expand it. The pGk13a, that is anchored to the gel matrix, is fluorescently labeled via click-chemistry (i.e., DBCO-fluorophore) at room temperature, overnight. (**a.vii**) Then the sample is incubated with a primary antibody at ~4 ºC, for 48-72 hours. (**a.vii**) Then the sample is incubated with a secondary antibody at ~4 ºC, for 48-48 hours. (**a.ix**) The sample is expanded with water at room temperature for 1.5 hours (exchanging water every 30 minutes).

a.

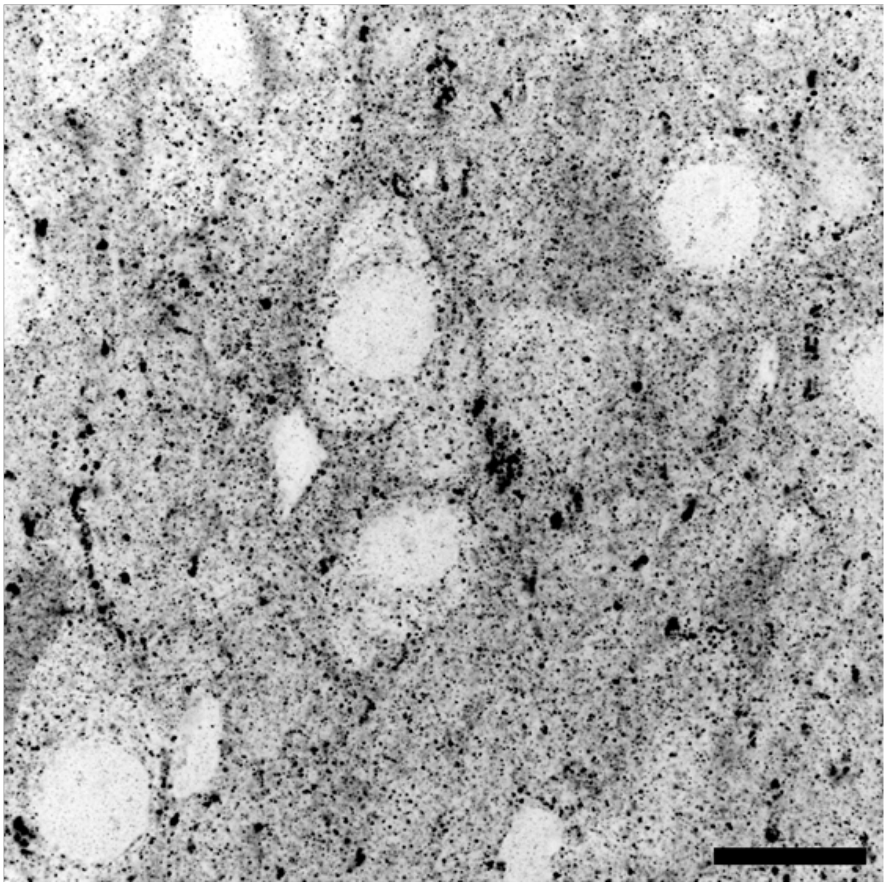

Supplementary Figure 22

(**a**) Representative (n=2 fixed brain slices from one mouse) single z-plane confocal image of expanded mouse brain tissue that underwent umExM protocol with the GMA anchor instead of AX anchor, showing pGk13a staining of the membrane in the hippocampus dentate gyrus. Scale bars are provided in biological units (i.e., physical size divided by expansion factor): (**a**) 10 µm

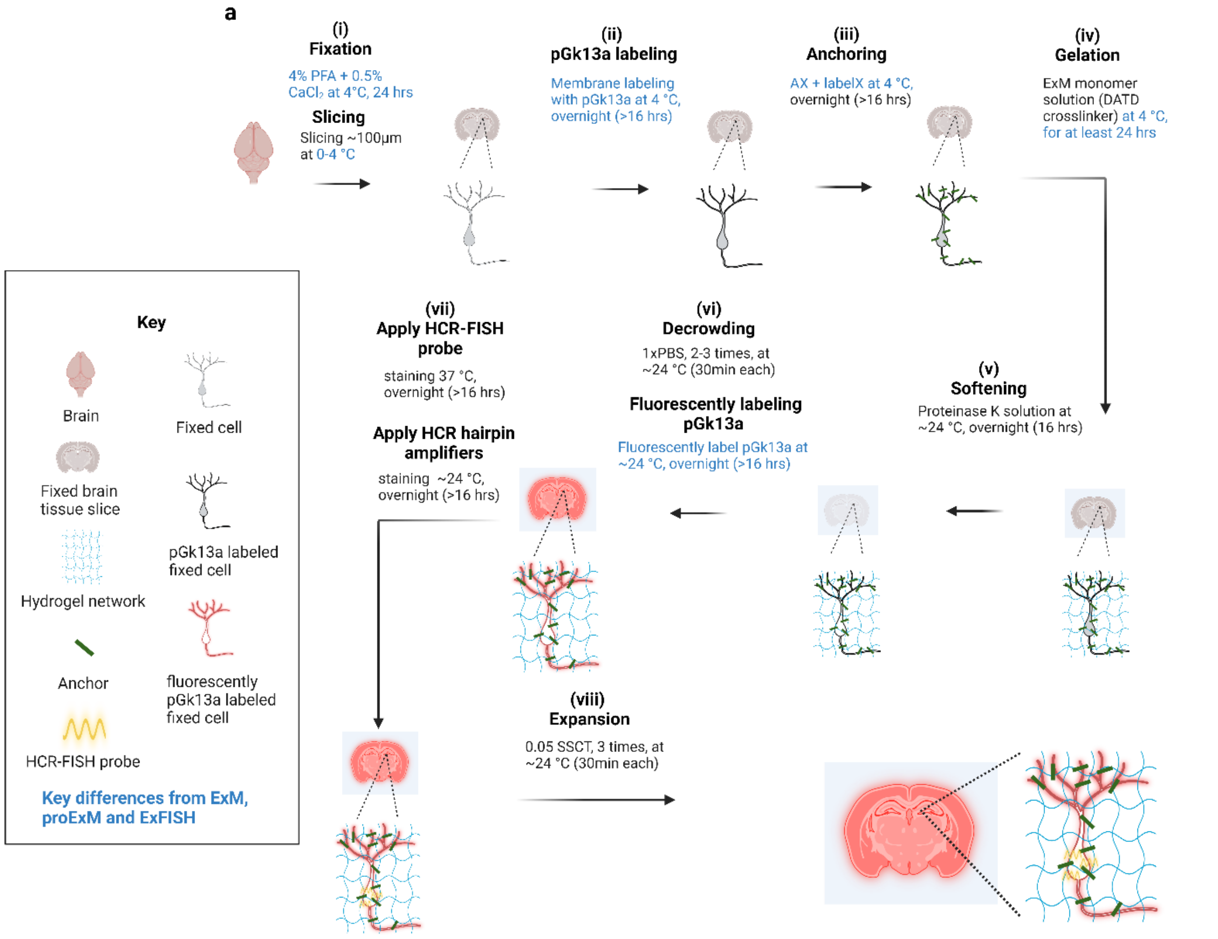

Supplementary Figure 23

(**a**) umExM with FISH workflow schematic. Blue-colored captions highlight the key differences from ExM[7], proExM[8] and ExFISH[25], whereas black captions highlight steps similar to those of ExM, proExM and ExFISH. (**a.i**) A specimen is chemically fixed with 4% paraformaldehyde (PFA) + 0.5% calcium chloride ($CaCl_2$) at 4 ºC for 24 hours. The brain is sliced on a vibratome at 100 µm thickness at 4 ºC. (**a.ii**) The specimen is treated with the pGk13a at 4 ºC overnight (unless otherwise noted, overnight means >16 hours throughout the figure). (**a.iii**) The specimen is treated with acrylic acid N-hydroxysuccinimide ester (AX) at 4 ºC overnight. (**a.iv**) The specimen is embedded in an expandable hydrogel (made with DATD crosslinker[15]) at 4 ºC for at least 24 hours. (**a.v**) The specimen is mechanically softened with enzymatic cleavage of proteins (i.e., specific cleavage with Proteinase k) at room temperature (~24 ºC), overnight. (**a.vi**) Then, the specimen-embedded hydrogel is treated with PBS to partially expand it. The pGk13a, that is anchored to the gel matrix, is fluorescently labeled via click-chemistry (i.e., DBCO-fluorophore) at room temperature, overnight. (**a.vii**) Then, specimen-embedded hydrogel is incubated with HCR-FISH probe at 37 ºC, overnight. (**a.vii**) Then, specimen-embedded hydrogel is incubated with fluorescently labeled HCR-hairpin amplifiers at ~24 ºC, overnight. (**a.viii**) The specimen-embedded hydrogel is expanded with 0.05x SSCT at room temperature for 1.5 hours (exchanging water every 30 minutes).

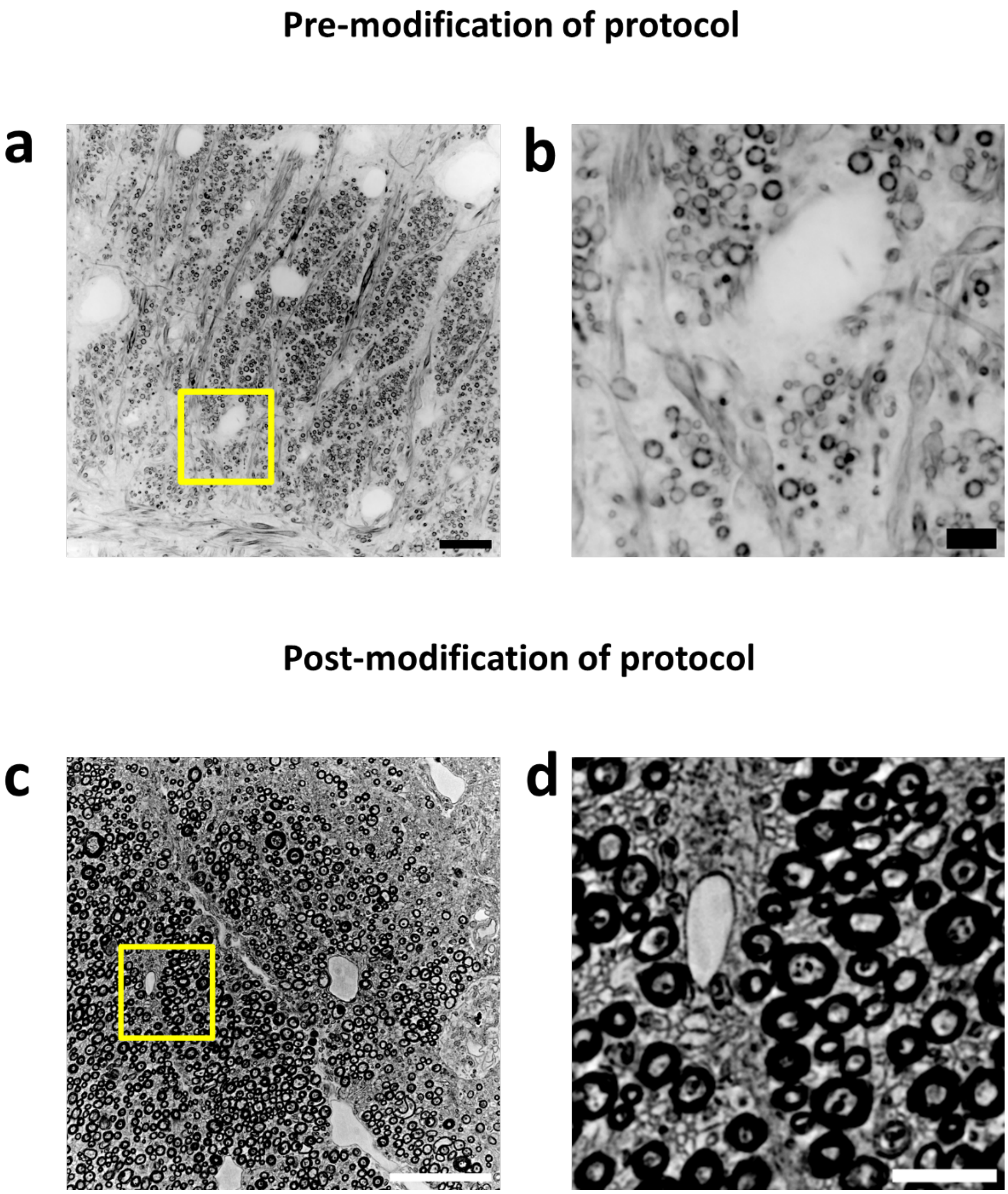

Supplementary Figure 24

(**a**) Representative (n=5 brain tissue sections from two mice) single z-plane confocal image of expanded mouse brain tissue after umExM protocol processing, showing pGk13a staining of the membrane in the corpus callosum region. The image is visualized in inverted gray color. (**b**) Magnified view of yellow boxed region in (**a**). Only a subset of axons can be identified in the images. (**c**) Representative (n=5 brain tissue sections from 2 mice) single z-plane confocal image of expanded mouse brain tissue after modified umExM protocol processing, showing pGk13a staining of the membrane in the corpus callosum region. The image is visualized in inverted gray color. (**d**) Magnified view of yellow boxed region in (**c**). The modification of the protocol drastically improved the visualization of axons in the corpus callosum. Scale bars are provided in biological units (i.e., physical size divided by expansion factor): (**a,c**) 10 μm, (**b**,**d**) 2 μm.

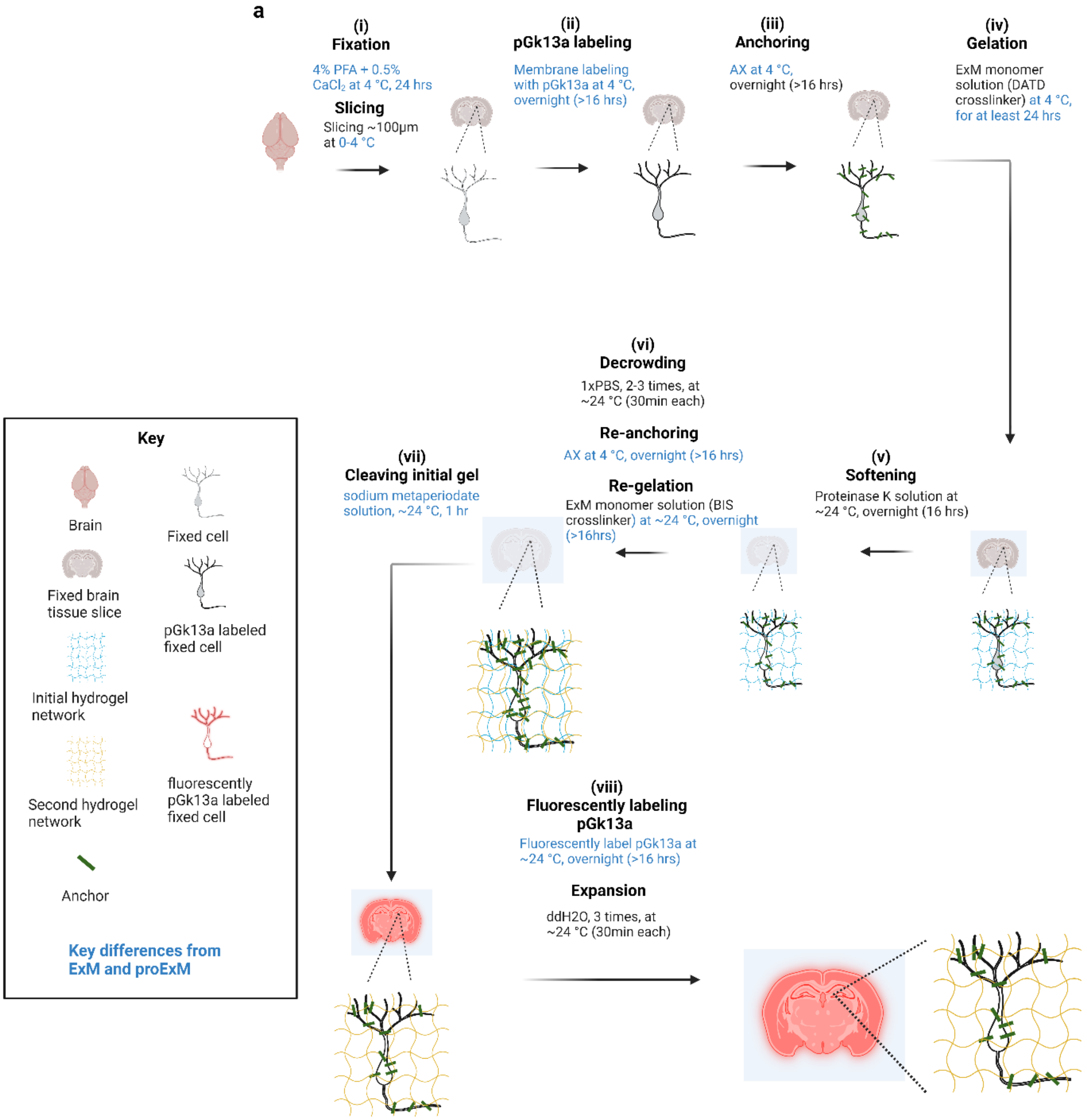

Supplementary Figure 25

(**a**) umExM with double gelation workflow schematic. Blue-colored captions highlight the key differences from ExM[7] and proExM[8], whereas black captions highlight steps similar to those in ExM and proExM. (**a.i**) A specimen is chemically fixed with 4% paraformaldehyde (PFA) + 0.5% calcium chloride ($CaCl_2$) at 4 ºC for 24 hours. The brain is sliced on a vibratome at 100 μm thickness at 4 ºC. (**a.ii**) The specimen is treated with pGk13a at 4 ºC overnight (unless otherwise noted, overnight means >16 hours throughout the figure). (**a.iii**) The specimen is treated with acrylic acid N-hydroxysuccinimide ester (AX) at 4 ºC overnight. (**a.iv**) The specimen is embedded in an expandable hydrogel (made with cleavable crosslinker DATD) at 4 ºC for at least 24 hours. (**a.v**) The specimen is mechanically softened with enzymatic cleavage of proteins (i.e., specific cleavage with proteinase-k) at room temperature (~24 ºC), overnight. (**a.vi**) The specimen-

embedded hydrogel is treated with PBS to partially expand it. Next, the sample is treated with AX as we did in (**a.iii**). Subsequently, the sample is gelled again but with a monomer solution that contains the non-cleavable crosslinker N,N -Methylenebis(acrylamide) (BIS) at room temperature (~24 ºC), overnight. (**a.vii**) Then the specimen-embedded hydrogel is incubated in a gel cleaving solution (containing sodium metaperiodate) at room temperature (~24 ºC) for 1 hour. This step cleaves the initial gel that was formed in (**a.iv**). (**a.viii**) Finally, the pGk13a, that is anchored to the gel matrix, is fluorescently labeled via click-chemistry (i.e., DBCO-fluorophore) at room temperature, overnight. The specimen-embedded hydrogel is expanded with water at room temperature for 1.5 hours (exchanging water every 30 minutes). The modified protocol requires additional time and resources (i.e., necessitating two gelations) compared to the unmodified protocol, which is suitable for all these regions except the corpus callosum. Hence, we suggest employing the modified protocol specifically for the corpus callosum region.

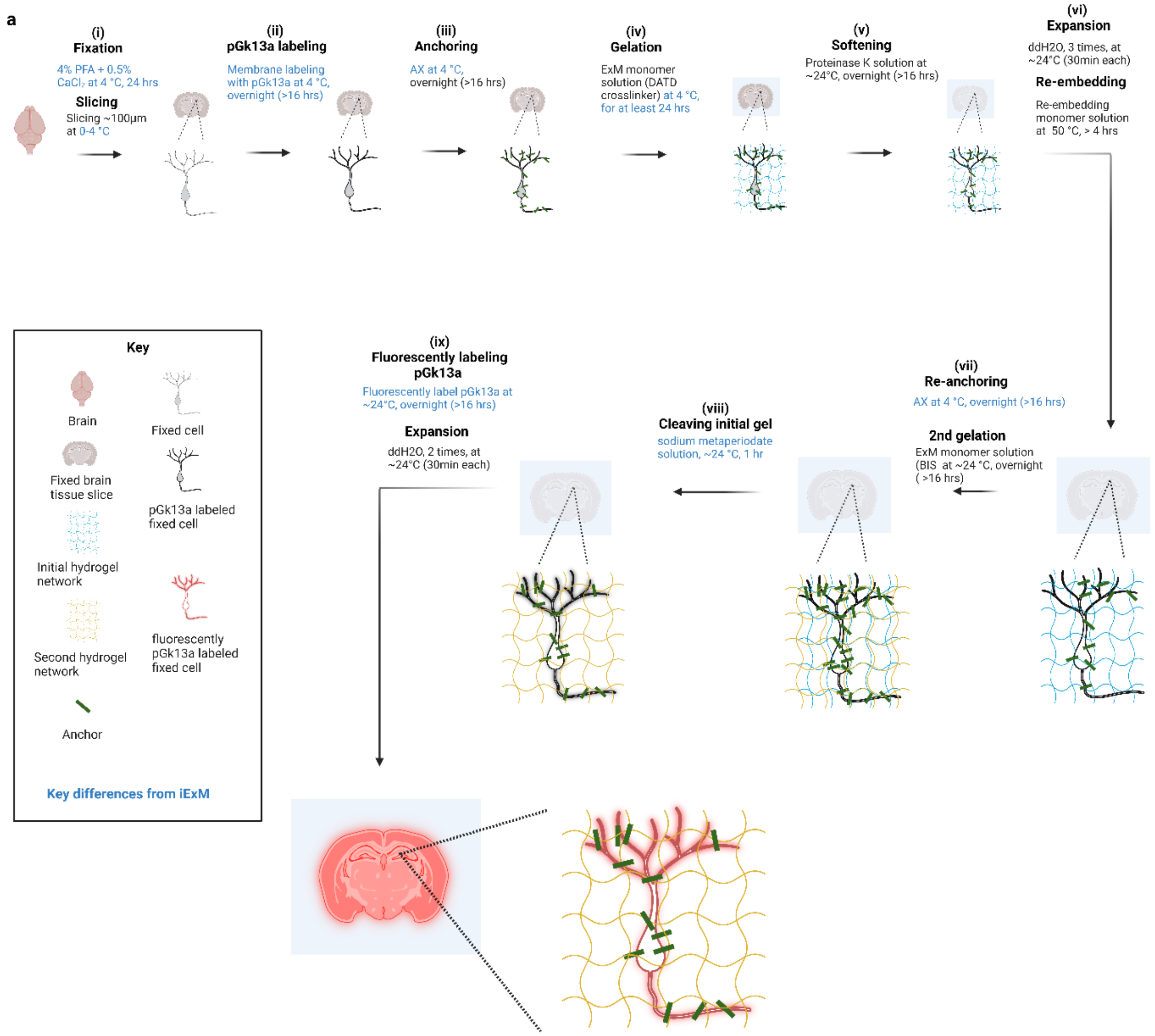

Supplementary Figure 26

(**a**) Iterative form of umExM workflow schematic. Blue-colored captions highlight the key differences from iExM[26], whereas black captions highlight steps similar to those in iExM. (**a.i**) A specimen is chemically fixed with 4% paraformaldehyde (PFA) + 0.5% calcium chloride ($CaCl_2$) at 4 ºC for 24 hours. The brain is sliced on a vibratome at 100 µm thickness at 4 ºC. (**a.ii**) The specimen is treated with pGk13a at 4 ºC overnight (unless otherwise noted, overnight means >16 hours throughout the figure). (**a.iii**) The specimen is treated with acrylic acid N-hydroxysuccinimide ester (AX) at 4 ºC overnight. (**a.iv**) The specimen is embedded in an expandable hydrogel (made with cleavable crosslinker DATD[24]) at 4 ºC for at least 24 hours. (**a.v**) The specimen-embedded hydrogel is mechanically softened with enzymatic cleavage of proteins (i.e., specific cleavage with proteinase-k) at room temperature (~24 ºC), overnight. (**a.vi**) The specimen-embedded hydrogel is expanded with water at room temperature for 1.5 hours (exchanging water every 30 minutes). Then, the specimen-embedded hydrogel is re-embedded into a non-expandable hydrogel at 50 ºC, for >4 hours. (**a.vii**) Next, the sample is treated with AX as we did in (**a.iii**). Subsequently, the sample is gelled again but with a monomer solution that

contains the non-cleavable crosslinker (made with BIS) at room temperature (~24 ºC), overnight. (**a.viii**) Then the specimen-embedded hydrogel is incubated in a gel cleaving solution (contains sodium metaperiodate) at room temperature (~24 ºC) for 1 hour. This step cleaves the initial gel and re-embedding gel that was formed in (**a.iv**) and (**a.vi**). (**a.ix**) Finally, the pGk13a, that is anchored to the gel matrix, is fluorescently labeled via click-chemistry (i.e., DBCO-fluorophore) at room temperature, overnight. The specimen-embedded hydrogel is expanded with water at room temperature for 1.5 hours (exchanging water every 30 minutes).

a

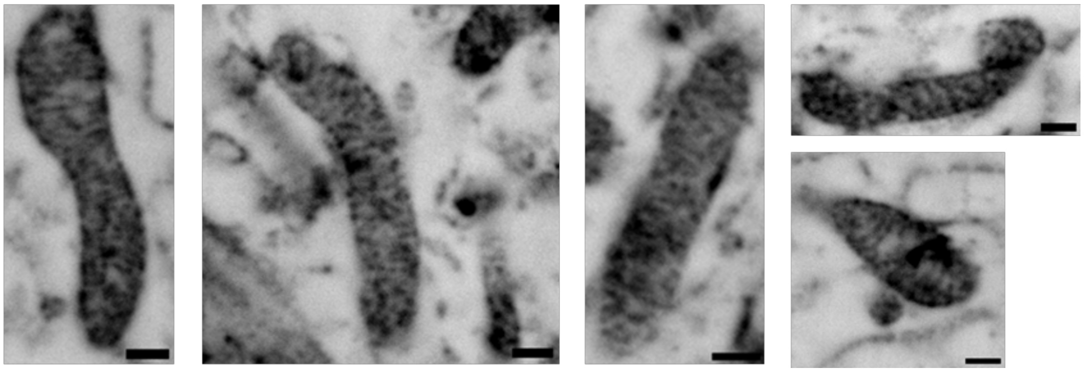

Supplementary Figure 27

(**a**) Five examples of single z-plane confocal images of post-expansion mouse brain tissue (somatosensory cortex, L4) after the iterative form of umExM processing, showing pGk13a staining of the mitochondrial cristae. Scale bars are provided in biological units (i.e., physical size divided by expansion factor): (**a**) 0.2 µm.

Supplementary Tables

Supplementary Table 1

The comparison table excludes TREx[23], LExM[27], TRITON ExM[28], uniExM[22], sphingolipid ExM[29], and TT-ExM[21] as they didn't show membrane or lipid labeling in tissue.

"N/A" denotes not available
* Measured with 100% laser and 100ms exposure time and 60x, 1.27NA water immersion lens

	Protocol	**umExM +mExM**	**Magnify**	**pan-ExM-t**	**clickExM**
**Protocol**	*membrane (lipid) labeling applied to mouse brain tissue?*	Yes	Yes	Yes	Yes
**Protocols for mouse brain tissue**	*whether it can be used with conventional fixatives*	Yes	Yes	Yes	No, requires acute brain slice
	which probe?	pGk13a	DiD	pacSph	alkyne-choline
	cost of the probe? (as of Oct. 2023)	$150/1mg	$5/1mg	$791/1mg	$15/1mg
	Can clearly visualize plasma membrane?	Yes	No	No	No
	What other membrane it can visualize?	-Mitochondria membrane<br>-Nuclear membrane<br>-Ciliary membrane<br>-Extracellular vesicle membrane<br>-Myelin sheaths	-Mitochondria membrane<br>-Bloodvessel membrane<br>-Myelin sheaths	-ER membrane<br>-Mitochondria membrane<br>-Nuclear membrane<br>-Myelin sheaths	unclear
	Claimed ultrastructure is preserved?	Yes	No	Yes	No
	How they validate ultrastructure preservation?	Quantitatively comparing the diameter of axon and cilia to previously reported values from EM and STED	N/A	Quantitatively comparing extracellular space content to previously reported values from STED	N/A
	Thickest tissue applied?	100µm	80µm	100µm	150µm
	Which brain region?	Cortex<br>Hippocampus<br>3rd ventricle<br>Choroid plexus<br>Corpus Callosum	unclear	Cortex<br>Hippocampus	Cortex
	*signal-to-background of the membrane signal**	~80	didn't measured	didn't measured	didn't measured

	continuity analysis performed?	Yes	No	No	No
	Post-expansion staining of proteins?	Yes	Yes	Yes	No
	Post-expansion staining of RNAs?	Yes	Yes	No	No
	How many antibodies are demonstrated to be used at once?	1	2	2	0
	How many steps involved to reach the highest claimed expansion	-1 membrane labeling step<br>-2 anchoring steps<br>-3 gelation steps<br>-1 softening step<br>-1 cleaving step<br>-2 expansion step	-1 membrane labeling step<br>-1 anchoring step<br>-1 gelation step<br>-1 softening step<br>-1 expansion step	-1 membrane labeling step<br>-1 anchoring step<br>-3 gelation steps<br>-1 softening step<br>-1 cleaving step<br>-2 expansion steps	-1 membrane labeling step<br>-1 anchoring step<br>-1 gelation step<br>-1 softening step<br>-1 expansion step
**Resolution**	*Claimed highest expansion factor*	12	11	24	4.5
	Claimed the highest resolution with conventional microscopy	35	didn't measure	didn't measure	didn't measure
	How do they measure the resolution?	Fourier-ring correlation	N/A	N/A	N/A
**Utility**	Segmentation of the cell body?	Yes	No	No	No
	Segmentation of the dendrite?	Yes	No	No	No
	Segmentation of the axon?	Yes, down to roughly 200nm diameter axons	No	No	No
	Tracing of the axon?	Yes	No	No	No

Supplementary Table 2
mExM solutions

pGk5b stock solution (prepared at RT and immediately stored at -20 ⁰C):

Reagent	Amount	Final concentration
pGk5b	1mg	10mg/1ml
Anhydrous DMSO (Thermo Fisher, cat. no. D12345)	50µl	
Water (Thermo Fisher, cat. no. 10977015)	50µl	
**Total**	**100µl**	

pGk5b membrane labeling stock solution (prepared fresh and used immediately at 4 ⁰C)

Reagent	Stock concentration	Amount (ml)
pGk5b stock solution (see above)	10mg/1ml	0.01
PBS (Corning, cat. no. 21031CM)*		0.99
**Total**		**1**

* Chilled on ice before use

AcX stock solution (prepared fresh and used immediately at 4 ⁰C*)

Reagent	Stock concentration	Amount (ml)
Acryloyl-X SE (Thermo Fisher, cat. no. A20770)	10mg/ml in DMSO**	0.01
PBS (Corning, cat. no. 21031CM)		0.99
**Total**		**1**

* Aliquot 20ul into a PCR tube, and store at -20 ⁰C in a sealed container (e.g., 50mL tube) with drying agents (e.g., Drierite)
** Anhydrous DMSO (Thermo Fisher, cat. no. D12345)

Monomer solution aka StockX (9.4ml, aliquoted to 10 tubes of 940µl and stored at -20 ⁰C):

Reagent	Stock concentration*	Amount (ml)
Sodium Acrylate (Sigma, cat. no. 408220)	38	2.25
Acrylamide (Sigma, cat. no. A8887)	50	0.5
N,N′-Methylenebisacrylamide (Sigma, cat. no. M7279)	2	0.75
Sodium chloride (Thermo Fisher, cat. no. BP358-212)	29.2	4
10xPBS (Thermo Fisher, cat. no. 70011044)	10x	1
Water (Thermo Fisher, cat. no. 10977015)		0.9
**Total**		**9.4**

*All concentrations are in g/100 ml except 10xPBS. All stock solutions are formulated in water (Thermo Fisher, cat. no. 10977015).

Gelling solution (1ml, prepared at 4 ⁰C, gelled at 37 ⁰C):

Reagent	Stock concentration*	Amount (µl)
Monomer Solution (see above)	1x	940

4-hydroxy-TEMPO (Sigma, cat. no. 176141)	0.5	20
TEMED (Sigma, cat. no. T7024)	10	20
APS (Thermo Fisher, cat. no. 17874)	10	20
**Total**		**1000**

*All concentrations are in g/100 ml except Monomer Solution. All stock solutions are formulated in water (Thermo Fisher, cat. no. 10977015).

To make the gelling solution, add 20µl of 4-hydroxy-TEMPO solution (0.005g/ml in water) and 20µl of TEMED solution (0.1g/ml in water) to 940µl of monomer solution, vortex for 2-3 seconds, add 20µl of APS solution (0.1g/ml in water), vortex for 2-3 seconds.

Digestion buffer* (100ml, prepared and stored at RT, applied at 37 ºC):

**Reagent**	**Stock concentration**	**Amount**
Tris pH 8.0 (Thermo Fisher, cat. no. AM9856)	1M	5ml
EDTA (Thermo Fisher, cat. no. 15575020)	0.5M	0.2ml
Triton X-100 (Sigma, cat. no. X100)	10%	5ml
NaCl (Sigma, cat. no. S5886)	>99% solid	5.85g
Water (Thermo Fisher, cat. no. 10977015)		84ml
**Total**		**100ml**

To formulate the Digestion solution, dilute Proteinase-K (NEB, cat. no. P8107S) at 1:100 dilution in Digestion buffer. All stock solutions are formulated in water (Thermo Fisher, cat. no. 10977015).

Fixation Reversal buffer (10ml, prepared at RT and used immediately):

**Reagent**	**Stock concentration***	**Amount**
PEG20000 (Sigma, cat. no. 95172-250G-F)	5%	1ml
DTT (Thermo Fisher, cat. no. R0862)	>97% solid	154.3mg
SDS (Thermo Fisher, cat. no. AM9820)	20%	2ml
Tris pH8 (Thermo Fisher, cat. no. AM9856)	1M	1ml
Water (Thermo Fisher, cat. no. 10977015)		5.9ml
**Total**		**10ml**

*All stock solutions are formulated in water (Thermo Fisher, cat. no. 10977015).

List of antibodies used for mExM:

**Antigen**	**Species**	**Company**	**Catalog no**	**Note**
**Tom20**	Rabbit	CST*	42406S	Identified as Vendor1 in Supplementary Fig. 7d, mitochondria bar
**Tom20**	Mouse	SCBT**	sc-17764	Identified as Vendor2 in Supplementary Fig. 7d, mitochondria bar
**Nup98**	Rabbit	CST*	2597S	
**MBP**	Rabbit	CST*	78896S	Identified as Vendor1 in Supplementary Fig. 7d, myelin bar
**MBP**	Rabbit	Abcam	ab40390	Identified as Vendor2 in Supplementary Fig. 7d, myelin bar
**MBP**	Chicken	AVES	AB_2313550	Identified as Vendor3 in Supplementary Fig. 7d, myelin bar

* Cell Signaling Technology
**Santa Cruz Biotechnology

Supplementary Table 3
umExM solutions

Fixative solution (prepared fresh and used immediately):

Reagent	Stock concentration	Amount
PFA (Electron Microscopy Sciences, cat. no. 15710)	16%	10ml
$CaCl_2$ (Millipore Sigma, cat. no. C4901)	≥97% solid	0.2g
Sodium cacodylate buffer, pH 7.4 (Electron Microscopy Sciences, cat. no. 11653)*	200mM	30ml
**Total****		40ml

*Chilled on ice before use
**Kept on ice during perfusion

pGk13a stock solution (prepared at RT and immediately stored at -20 ^{0}C):

Reagent	Amount	Final concentration
pGk13a	1mg	11.1mg/1ml
Anhydrous DMSO (Thermo Fisher, cat. no. D12345)	45µl	
Water (Thermo Fisher, cat. no. 10977015)	45µl	
**Total**	**90µl**	

pGk13a membrane labeling stock solution (prepared fresh and used immediately at 4 ^{0}C)

Reagent	Stock concentration	Amount (ml)
pGk13a stock solution (see above)	11.1mg/ml	0.015
PBS (Corning, cat. no. 21031CM)*	1x	0.985
**Total**		**1**

* Chilled on ice before use

AX buffer solution (prepared fresh and stored at 4 ^{0}C)

Reagent	Amount	Final concentration
MES (Sigma, cat. no. M3058)	0.434mg	0.4344g/20ml
5M Sodium chloride (Thermo Fisher, cat. no. BP358-212)	0.6ml	
1M HCl	1ml	
Water (Thermo Fisher, cat. no. 10977015)*	18.4ml	
**Total**	20ml	

* Chilled on ice before use

AX stock solution (prepared fresh and used immediately at 4 ^{0}C, and stored at -20 ^{0}C*)

Reagent	Stock concentration	Amount (ml)
Acrylic acid N-hydroxysuccinimide ester (Thermo Fisher, cat. no. AC400300010)	10mg/ml in DMSO**	0.006
AX buffer solution (see above)		0.994
**Total**		**1**

* Aliquot 20ul into a PCR tube, and store at -20 ^{o}C in a sealed container (e.g., 50mL tube) with drying agents (e.g., Drierite)
** Anhydrous DMSO (Thermo Fisher, cat. no. D12345)

umExM monomer solution (9.4ml, aliquoted to 10 tubes of 940µl and stored at -20 ^{o}C):

Reagent	Stock concentration*	Amount (ml)
Sodium Acrylate (Sigma, cat. no. 408220)	38	2.25
Acrylamide (Thermo Fisher, cat. no. 15512023)	50	0.5
N,N'-Diallyl-L-tartardiamide (Alfa Aesar, cat. no. A12195-30)	9	0.75
5M Sodium chloride (Thermo Fisher, cat. no. BP358-212)	29.2	4
10xPBS (Thermo Fisher, cat. no. 70011044)	10x	1
Water (Thermo Fisher, cat. no. 10977015)		0.9
**Total**		**9.4**

*All concentrations are in g/100 ml except 10xPBS.

umExM gelling solution (1ml, prepared and gelled at 4 ^{o}C):

Reagent	Stock concentration*	Amount (µl)
umExM monomer Solution (see above)	1x	940
4-hydroxy-TEMPO (Sigma, cat. no. 176141)	0.5	20
TEMED (Sigma, cat. no. T7024)	10	20
APS (Thermo Fisher, cat. no. 17874)	10	20
HCl	1M	14
**Total**		**1014****

*All concentrations are in g/100 ml except umExM Monomer Solution. All stock solutions are formulated in water (Thermo Fisher, cat. no. 10977015).
**To make umExM gelling solution, add 20µl of 4-hydroxy-TEMPO solution (0.005g/ml in water) and 20µl of TEMED solution (0.1g/ml in water) to 940µl of umExM monomer Solution, vortex for 2-3 seconds, add 20µl of APS solution (0.1g/ml in water), vortex for 2-3 seconds, and add 4µl of 1M HCl, and vortex for 2-3 second.

umExM Digestion buffer* (100ml, prepared and applied at RT, and stored at 4 ^{o}C):

Reagent	Stock concentration	Amount
Tris pH 8.0 (Thermo Fisher, cat. no. AM9856)	1M	5ml
EDTA (Thermo Fisher, cat. no. 15575020)	0.5M	0.2ml
Saponin (Sigma, cat. no. 84510)	10%	5ml
5M Sodium chloride (Thermo Fisher, cat. no. BP358-212)	5M	
Water (Thermo Fisher, cat. no. 10977015)		84ml
**Total**		**100ml**

*To formulate the Digestion solution, dilute Proteinase-K (NEB, cat. no. P8107S) at 1:100 dilution in Digestion buffer. All stock solutions are formulated in water (Thermo Fisher, cat. no. 10977015).

Trypsin+Lys-C softening solution (2ml, prepared fresh and applied at RT):

Reagent	Amount	Final concentration

Trypsin/Lys-C (Thermo Fisher, cat. no. A40007)	20µg	20µg/2ml
TRIS (1M), pH 8.0 (Thermo Fisher, cat. no. AM9855G)	0.1ml	
PBS (Corning, cat. no. 21031CM)	1.9ml	
**Total**	**2ml**	

Second monomer solution (34.5ml, prepared fresh):

Reagent	Stock concentration*	Amount (ml)
Acrylamide (Thermo Fisher, cat. no. 15512023)	50	10
N,N'-Diallyl-L-tartardiamide (Alfa Aesar, cat. no. A12195-30)	5	5
Water (Thermo Fisher, cat. no. 10977015)		0.9
**Total**		**34.5**

*All concentrations are in g/100 ml.

Second gelling solution (50ml, prepared fresh):

Reagent	Stock concentration*	Amount (ml)
Second monomer solution	1x	34.5
N,N'-Diallyl-L-tartardiamide (Alfa Aesar, cat. no. A12195-30)	5	5
TEMED (Sigma, cat. no. T7024)	10	0.25
APS (Thermo Fisher, cat. no. 17874)	10	0.25
**Total**		**50**

*All concentrations are in g/100 ml except cleavable second monomer solution.

To make the second gelling solution, 250µl of TEMED solution (0.1g/ml in water) to 34.5mL of second monomer solution, vortex for ~10 seconds, add 250µl of APS solution (0.1g/ml in water), vortex for ~10 seconds.

Third monomer solution (9.4ml, aliquoted to 10 tubes of 940µl and stored at -20 ^{0}C):

Reagent	Stock concentration*	Amount (ml)
Sodium Acrylate (Sigma, cat. no. 408220)	38	2.25
Acrylamide (Sigma, cat. no. A8887)	50	0.5
N,N′-Methylenebisacrylamide (Sigma, cat. no. M7279)	2	1
Sodium chloride (Thermo Fisher, cat. no. BP358-212)	29.2	4
10xPBS (Thermo Fisher, cat. no. 70011044)	10x	1
Water (Thermo Fisher, cat. no. 10977015)		0.65
**Total**		**9.4**

*All concentrations are in g/100 ml except 10xPBS.

Third gelling solution (prepared in fresh and applied at RT):

Reagent	Stock concentration*	Amount (µl)
umExM monomer Solution (see above)	1x	940
4-hydroxy-TEMPO (Sigma, cat. no. 176141)	0.5	20
TEMED (Sigma, cat. no. T7024)	10	20
APS (Thermo Fisher, cat. no. 17874)	10	20
**Total**		**1000****

*All concentrations are in g/100 ml.
** To make the third gelling solution, add 20µl of 4-hydroxy-TEMPO solution (0.005g/ml in water) and 20µl of TEMED solution (0.1g/ml in water) to 940µl of monomer solution, vortex for 2-3 seconds, add 20µl of APS solution (0.1g/ml in water), vortex for 2-3 seconds.

List of antibodies used for umExM:

Antigen	Species	Company	Catalog no
**SV2A**	Rabbit	Abcam	50-194-3924
**PSD95**	Rabbit	Thermo Fisher	MA1-046

Supplementary Table 4

We measured the laser excitation power (mW). To do so, we employed a Nikon W1 spinning disk equipped with a four-line laser system. Since we utilized the 561nm laser line for pGk13a signals, and we only reported exposure time for this one laser line, we measured the laser excitation power (mW) of this line. This measurement was performed using a power meter to directly measure the excitation light output from the 4x, 10x, 40x and 60x objective lenses that were used for imaging pGk13a signals throughout:

Lens	Laser power	
	**50%**	**100%**
4x	1.10 mW	2.33mW
10x	1.11mW	2.35mW
40x	0.97mW	2.02mW
60x	0.94mW	2.00mW

Supplementary Methods

Electron microscope imaging, visualization, and analysis

To validate pGk5b labeling by electron microscopy (EM), tissue slices of 100 µm thickness were treated with lipid labeling solution as we used for mExM, except we used azide instead of biotin as the linkable group of the lipid stain (pGk5a for short): we first incubated the tissue in 1ml of pGk5 (0.1µg/ml in ice-cold PBS) at 4 ºC overnight (>16hrs) to let the labels diffuse and intercalate thoroughly throughout. Subsequently, the sample was washed 2x using PBS at 4 ºC for 1 hour each to remove any excess lipid label. Then the sample was placed in 2% PFA, 2% glutaraldehyde in PBS at 4 ºC for 6 hours for post-fixation of the sample for EM staining. This fix also served for further EM processing, to preserve the state of ultrastructure[1,2]. These steps were performed at 4 ºC to promote the stability of the lipids and the lipid label in the sample. The sample was moved to 1.8nm undecagold-DBCO conjugate solution (2.5mg/1mL Nanopartz, part no. CK11) at 4 ºC for 12 hours. The azide-DBCO chemistry served to link the lipid label with a gold nanoparticle. Thereafter the sample was washed 3x with 0.15M sodium cacodylate buffer at room temperature for 30 minutes each to remove unbound nanoparticles. The samples were sent to the Harvard Medical School Electron Microscopy Core to be stained, then embedded and sliced using a standard EM preparation protocol[2]. In summary, the tissue was stained with 1% uranyl acetate (UA) for 1 hour at room temperature, embedded in resin, and sliced in ultrathin sections (40nm thickness). As discussed in **Supplementary Fig. 1**, we decided to use a common UA staining protocol (1% UA for 1 hour at RT) to enhance the pGk5a signals on top of signals from gold nanoparticles as UA can react to amino groups of pGk5a. As for control experiments, the protocol was adjusted by replacing pGk5a staining with common osmium staining (1% OsO4

for 1 hour at RT). Samples were imaged on a JEOL 1200EX transmission electron microscope using 80keV transmitted voltage. The images were captured with an AMT 2k CCD camera. Acquired images were processed with a Gaussian filter (Radius = 1) and Enhance contrast (Saturated pixels = 10.5%) function in ImageJ (version 1.53q). pGk5a treated sample without OsO_4 clearly showed membranes (e.g., mitochondrial membrane and vesicle membranes; **Supplementary Fig. 1a**) similar to the control experiment (i.e., that is, only with OsO_4; **Supplementary Fig. 1b**) but with slightly lower contrast.

Cell preparation

We first inserted a 13-mm-diameter coverslip (Thermo Fisher, catalog no. 174950) into one well of a 24-glass well plate (Cellvis 24 WELL GLASS BTTM PLATE 20/CS, catalog no. NC0397150). Then, either HEK293 or HeLa or U2OS cells were plated in the well (~40k cells/ml in cell culture medium (described in next paragraph) per well.) The plate was then moved to a humidified cell culture incubator (set at 37°C, 20% oxygen, and 5% CO2) for at least 6 hours for cells to adhere. The cells were fixed with 4% paraformaldehyde (PFA) and 0.1% glutaraldehyde in Dulbecco's 1x phosphate buffered saline (PBS) at room temperature (RT) for 15 minutes. Fixed cells were washed 4 times with PBS for 10 minutes each at 4 ^{o}C, and kept in PBS at 4 ^{o}C.

For HEK293 cell culture medium, we used Dulbecco’s modified Eagle’s medium (DMEM, Corning, catalog no. 10013CV) supplemented with 10% heat-inactivated fetal bovine serum (FBS, Thermo Fisher, catalog no. A3840001), 2 mM GlutaMax (Thermo Fisher Scientific, cat. No. 3505006), and 1% penicillin-streptomycin (Thermo Fisher, catalog no. 15140122). For HeLa

cell culture medium, we used DMEM supplemented with 10% heat-inactivated fetal bovine serum (FBS) and 1% penicillin-streptomycin (Thermo Fisher, catalog no. 15140122). For U2OS cell culture medium, we used Dulbecco's modified Eagle's medium (DMEM, Corning, catalog no. 10013CV) supplemented with 10% heat-inactivated fetal bovine serum (FBS, Thermo Fisher, catalog no. A3840001), 1% penicillin-streptomycin (Thermo Fisher, catalog no. 15140122) and 1% sodium pyruvate (Thermo Fisher, catalog no. 11360070).

Transduction of cells via BacMam virus

The adherent cells were prepared as described in the Cell Preparation section. The cells were transduced by directly adding 12µl of BacMam reagent (either CellLight™ Mitochondria-GFP, catalog no. C10508 or CellLight™ ER-GFP, catalog no. C10590) to the cell medium. The cells were then placed in the culture incubator overnight (>16hrs). The cells were then fixed and washed as described in **the Cell Preparation section** in **Supplementary Methods**.

Brain tissue preparation for mExM

All procedures involving animals were in accordance with the US National Institutes of Health Guide for the Care and Use of Laboratory Animals and approved by the Massachusetts Institute of Technology Committee on Animal Care. Wild type (both male and female, C57BL/6 or Thy1-YFP, 6-8 weeks old, from either Taconic or JAX) mice were first terminally anesthetized with isoflurane. Then, ice-cold PBS was transcardially perfused until the blood cleared (approximately 25ml). For all mExM experiments, the mice were then transcardially perfused with 4% paraformaldehyde (PFA) and 0.1% glutaraldehyde in ice-cold PBS. The fixative was

kept on ice during perfusion. After the perfusion step, brains were dissected out, stored in fixative at 4 ºC for 12 hours for further fixation, and sliced on a vibratome (Leica VT1000S) at 100 µm thickness. For the slicing, the tray was filled with ice-cold PBS, and the tray was surrounded by ice. The slices were kept in PBS at 4 ºC overnight for washing and storing.

mExM for cells

1. The fixed cells (as described in the **Cell Preparation** section in **Supplementary Methods**) were incubated in the pGk5b solution (**Supplementary Table 2,** "pGk5b membrane labeling stock solution") at 4 ºC overnight.
2. The cells were then incubated in the AcX solution (**Supplementary Table 2,** "AcX stock solution") overnight at 4 ºC. Then we washed with ice-cold PBS 2 times, 30min each at 4 ºC.
3. The cells were then incubated in the gelling solution (**Supplementary Table 2, "**Gelling solution") 30min at 4 ºC for pre-gelation. During this step, the gelation chamber was constructed as described previously[3]. In summary, we placed two spacers (VWR, catalog no. 48368-085) on a microscope slide (VWR, catalog no. 48300-026). The spacers were separated from each other enough so that an adherent cell-containing cover glass could be placed in between them. The adherent cell-containing cover glass was then placed between the spacers on the slide. We then placed the lid (VWR, catalog no. 87001-918) on top of the spacers, covering the cell-containing cover glass. We then fully filled the empty space between the cells and spacers with the gelling solution. Next, the chamber was transferred to a 37 ºC incubator to initiate free-radical polymerization. After 2 hours, the gelation chamber containing cells was taken out

4. The gel was trimmed with a razor blade (VWR, cat. no. 55411-050) and transferred from the chamber to a 6-well plate (Thermo Fisher, catalog no. 140675) that contained proteinase K digestion buffer (**Supplementary Table 2, "**Digestion buffer") in the well (3mL of digestion buffer per well). The gel was then digested at 37C on a shaker overnight (> 16 hours). After digestion, the gel was washed 4 times in PBS at RT, 30 minutes each.
5. The digested gels were labeled with 0.3mg/ml of streptavidin labeled with Atto 565 (Atto 565-Streptavidin; Sigma Aldrich, catalog no. 56304-1MG-F) buffered in PBS overnight at RT, and then washed 4 times in PBS at room temperature (RT), 30 minutes each.
6. The gels were placed 4 times in excess water at RT for expansion, 30 minutes each.

mExM for brain tissue slices

1. The fixed tissue slices (as described in the **Brain tissue preparation for mExM** section in **Supplementary Methods**) were incubated in a lipid labeling solution (**Supplementary Table 2,** "Lipid labeling stock solution") at 4 ºC overnight (>16 hours) to let the labels diffuse and intercalate thoroughly throughout the tissue slices.
2. The tissue slices were then incubated in an AcX stock solution (**Supplementary Table 2,** "AcX stock solution") overnight (>16 hours) at 4 ºC. The tissue was then washed 2 times in PBS at 4 ºC, 1 hour each.
3. The tissue slices were then incubated in gelling solution (**Supplementary Table 2,** **"**Gelling solution") 30min at 4 ºC for pre-gelation incubation. During this step, the gelation chamber was constructed as previously described[7]. In summary, we placed two spacers (VWR, catalog no. 48368-085) on a microscope slide (VWR, catalog no. 48300-

026). The two spacers were separated from each other enough so that the brain tissue slice could be placed in between them. The brain tissue slice was placed between the spacers. We then placed the lid (VWR, catalog no. 87001-918) on top of the spacers as well as the brain tissue slice. We then fully filled the empty space between the brain tissue slice and spacers with the gelling solution. The chamber was transferred to a 37 ^{o}C incubator to initiate free-radical polymerization. After 2 hours, the gelation chamber containing the tissue was taken out.

4. The gel was trimmed with a razor blade (VWR, cat. no. 55411-050) and transferred from the chamber to a 6-well plate (Thermo Fisher, catalog no. 140675) that contained proteinase K digestion buffer (**Supplementary Table 2**, "Digestion buffer") in the well (4mL of digestion buffer per well). The gel was then digested at 37C on a shaker overnight (>16 hours). After digestion, the gel was washed 4 times in PBS at RT, 30 minutes each.
5. The digested gels were labeled with 0.3mg/ml of streptavidin labeled with Atto 565 (Atto 565-Streptavidin; Sigma Aldrich, catalog no. 56304-1MG-F) buffered in PBS overnight at RT, and then washed 4 times in PBS at room temperature (RT), 30 minutes each.
6. The gels were placed 4 times in excess water at RT for expansion, 30 minutes each.

Immunohistochemistry-compatible mExM

The aforementioned mExM steps were carried out the same, except for the digestion step (i.e., step4 in the mExM for cells and mExM for brain tissue slices sections). Instead of using the proteinase K digestion buffer, the sample was heated in fixation reversal (FR; Supp Table 1, "Fixation Reversal buffer") buffer for 30 minutes at 100 ^{o}C and then held for 2 hours at 80 ^{o}C.

The FR buffer consisted of 0.5% PEG20000, 100mM DTT, 4% SDS, in 100mM Tris pH8. After this, the FR-digested sample was washed in 1x PBS 4 times at RT for 1 hour before proceeding to the immunohistochemistry steps. The expanded gels were first blocked with MAXblock Blocking Medium (Active Motif, catalog no. 15252) for 4-6 hours at room temperature and incubated in MAXbind Staining Medium (Active Motif, catalog no. 15251) containing primary antibodies at a concentration of 10 μg/ml overnight at 4 ºC. Then, the sample was washed with MAXwash Washing Medium (Active Motif, catalog no. 15254) at RT 4 times, 30 minutes each and subsequently incubated in secondary antibodies buffered in MAXbind Staining Medium at a concentration of 10 μg/ml for 10-12 hours at 4 ºC. Finally, the secondary antibodies were washed, again, with MAXwash Washing Medium at RT 4 times, 30 minutes each time. For primary antibodies, anti-TOM20 (Cell Signaling Technology, catalog no. 42406S, rabbit; Santa Cruz Biotechnology, catalog no. sc-17764, mouse), anti-NUP98 (Cell Signaling Technology, catalog no. 2597S, rabbit), anti-myelin basic protein (MBP; Cell Signaling Technology, catalog no. 78896S, rabbit; Abcam, catalog no. ab40390, rabbit; AVES, catalog no. AB_2313550, chicken), were used. For secondary antibodies, anti-chicken Alexa Fluor Plus 488 (Thermo Fisher, catalog no. A32931), anti-rabbit Alexa Fluor Plus 488 (Thermo Fisher, catalog no. A32731), and anti-mouse Alexa Fluor Plus 647 (Thermo Fisher, catalog no. A32728) were used. After antibody staining, the pGk5b probes that were conjugated to the gel were then labeled with 0.3mg/ml of streptavidin labeled with Atto 565 (Atto 565-Streptavidin; Sigma Aldrich, catalog no. 56304-1MG-F) buffered in PBS overnight at RT, and then washed 4 times in PBS at RT, 30 minutes each. Finally, the gel was placed 4 times in excess water at RT for expansion, 30 minutes each.

Antibody staining of fluorescent proteins for mExM

The expanded samples, after either proteinase-k digestion or high-temperature softening, were incubated with MAXblock Blocking Medium (Active Motif, catalog no. 15252) for 4-6 hours at room temperature and incubated in MAXbind Staining Medium (Active Motif, catalog no. 15251) containing fluorophore-conjugated primary antibody against the green fluorescent protein (GFP) at a concentration of 10 μg/mL overnight (> 16 hours) at 4 ºC. For the primary antibody, we used anti-GFP (Thermo, catalog no. A-21311). Next, the sample was washed with MAXwash Washing Medium (Active Motif, catalog no. 15254) at RT 4 times, 30 minutes each. After antibody staining, the lipid labels that were conjugated to the gel were then labeled with 0.3mg/ml of streptavidin labeled with Atto 565 (Atto 565-Streptavidin; Sigma Aldrich, catalog no. 56304-1MG-F) buffered in PBS overnight at RT, and then washed 4 times in PBS at RT, 30 minutes each. Finally, the gel was placed 4 times in excess water at RT for expansion, 30 minutes each.

Expansion factor and degree of isotropy analysis

For umExM, expansion factor and degree of isotropy analysis were carried out with cells. mExM protocol for cells is described **mExM for cells** section in supplementary methods. umExM protocol for cells is similar as mExM for cells, but using the pGk13a membrane labeling stock solution for step 1, AX stock solution for step 2, umExM gelling solution for step 3, umExM Digestion buffer for step 4, and pGk13a was fluorescently labeled with 1ml of Cy3 conjugated DBCO (Cy3 DBCO Click chemistry tools, catalog no. A140-1) buffered in PBS at a concentration of 0.03mg/ml on the shaker (50 rpm) at RT, overnight (>16 hours). The samples were expanded with water.

We evaluated the expansion factor as previously described[7,8]. In particular, we used HEK293 and U2OS cells transfected with BacMam viruses expressing GFP proteins targeted to the matrix of mitochondria. We randomly chose two landmarks in pre-expansion images and found the corresponding landmarks in expanded-cell images, and calculated the ratio.

We evaluated distortion as previously described[7,8]. In summary, we used BacMam virus to express GFP proteins in the matrix of mitochondria in HEK293 and U2OS cells. We imaged the cell with SIM before expansion, and re-imaged the same region after expansion with a confocal microscope. We non-rigidly registered the SIM image and the confocal image, then calculated the root-mean-square (RMS) length measurement error as a function of measurement length for SIM vs. expanded-cell images.

For the iterative form of umExM, expansion factor was measured with slices of fixed mouse brain. We measured the gel size before and after expansion (i.e., after 2nd round of expansion) and divided the measured gel size to obtain expansion factor.

Colocalization analysis for mExM images

We performed a colocalization analysis for mExM by adopting recommended colocalization methods for light microscopy studies[30,31]. We first segmented the foreground and background fluorescence of GFP channels of mExM images using the Otsu image processing algorithm[32] as we previously did for segmenting signals[9,33]. We then created a binary signal mask based on the foreground signals and used the signal mask to segment the pGk5b signals. Finally, we evaluated the fraction of expressed GFP and antibody signals that had pGk5b signals by counting the pixels containing pGk5b signals that were above 1x standard deviation below the mean of the pGk5b

signal intensity in the image. The analysis was performed with RStudio 2021.09.2+382 with R version 4.1.2.

ExM with fGk5b and fGk5b+pGk5b.

We followed the mExM protocol described in the supplementary method section. For ExM with fGk5b, we used 10mM fGk5b, the same concentration as the membrane probe used in the mExM protocol (10mM pGk5b), and the rest of the procedure was identical to that of mExM. Similarly, for ExM with fGk5b + pGk5b, we used 5mM fGk5b + 5mM pGk5b or 10mM fGk5b + 10mM pGk5b, and for ExM with pGk5b, we used 10mM pGk5b, while the rest of the downstream protocol remained the same as mExM.

umExM with GMA anchoring

Our GMA anchoring protocol was adapted from the published protocol[22]. Briefly, we performed step 1 in the **umExM for brain tissue slices** section. We then used 0.1% GMA in 100 mM sodium bicarbonate on a shaker (~10-20 rpm) at room temperature, overnight. We then performed steps 3-6 in the **umExM for brain tissue slices** section.

Biotin-DHPE, BODIPY-lipid, mCling staining and proExM

Biotin-DHPE

The Biotin-DHPE followed by tyramide signal amplification protocol was adopted from ref[21]. In summary, 4% PFA fixed mouse brain tissue was incubated in biotin-DHPE solution (Biotium, catalog no. 60022; 0.1 mg/ml in 50% ethanol) overnight at RT. Premixed ABC solution was prepared by using equal amounts of avidin and biotin from the VECTASTAIN ABC-HRP Kit via mixing them in TBS at a 1:50 dilution and incubated for 30 minutes. During this time, biotin-

DHPE-tissue was extensively washed at RT with PBS to remove unbound biotin-DHPE. The premixed ABC solution was then added to the biotin-DHPE-labeled tissue for 1 hour, followed by extensive washing at RT with PBS. After washing, Alexa Fluor-555-conjugated tyramide solution (Invitrogen, catalog no. B40955) in TRIS (1:100 dilution) containing hydrogen peroxide (0.03%; note that ref[21] used amplification dilution buffer which is currently discontinued), was applied to the tissue for 20 minutes at RT, and followed by extensive washing at RT with PBS.

BODIPY-lipid

The BODIPY-FL-C12 (BODIPY) staining protocol was adopted from ref[22]. In summary, 4% fixed mouse brain tissue was incubated in 10 μg/mL BODIPY (Invitrogen, catalog no. D3822) in PBS at RT, overnight. BODIPY stained tissue was then extensively washed with PBS.

mCling

The mCling staining protocol was adopted from ref[23]. In summary, 4% fixed mouse brain tissue was incubated in 5 μM BODIPY (Synaptic Systems, catalog no. 710 006AT1) in PBS at RT, overnight. mCling stained tissue was then extensively washed with PBS.

proExM

After treating tissue with either biotin-DHPE, BODIPY, or mCling staining protocols, they were processed using the standard proExM[8] protocol. In brief, tissue was incubated in 0.1 mg/ml of AcX (Thermo Fisher, catalog no. A20770), then gelled with the gelling solution (see **Supplementary Table 2**), digested with the digestion solution (see **Supplementary Table 2**), and finally expanded with water.

IMAXT Consortium Members At Time of Work Performed

List of IMAXT Grand Challenge Consortium investigators:

Greg Hannon, Cancer Research UK Cambridge Institute
Shankar Balasubramanian, Cancer Research UK Cambridge Institute
Bernd Bodenmiller, University of Zurich
Carlos Caldas, Cancer Research UK Cambridge Institute
Dario Bressan, Cancer Research UK Cambridge Invstitute
Johanna Joyce, University of Lausanne
Nicholas Walton, University of Cambridge
Owen Harris, Suil Interative Ltd
Sam Aparicio, The University of British Columbia
Simon Tavare, Cancer Research UK Cambridge Institute
Xiaowei Zhuang, Harvard University
Ed Boyden, Massachusetts Institute of Technology
Sohrab Shah, The University of British Columbia

Supplementary References

1. Skepper, J. N. Immunocytochemical strategies for electron microscopy: choice or compromise. *J. Microsc.* **199**, 1–36 (2000).
2. Eltoum, I., Fredenburgh, J., Myers, R. B. & Grizzle, W. E. Introduction to the Theory and Practice of Fixation of Tissues. *J. Histotechnol.* **24**, 173–190 (2001).
3. Hobot, J. A. & Newman, G. R. Strategies for improving the cytochemical and immunocytochemical sensitivity of ultrastructurally well-preserved, resin embedded biological tissue for light and electron microscopy. *Scanning Microsc. Suppl.* **5**, S27-40; discussion S40-1 (1991).
4. Palay, S. L., McGEE-RUSSELL, S. M., Gordon, S. & Grillo, M. A. FIXATION OF NEURAL TISSUES FOR ELECTRON MICROSCOPY BY PERFUSION WITH SOLUTIONS OF OSMIUM TETROXIDE. *J. Cell Biol.* **12**, 385–410 (1962).
5. Schmidt, R. *et al.* Mitochondrial cristae revealed with focused light. *Nano Lett.* **9**, 2508–2510 (2009).

6. Stephan, T., Roesch, A., Riedel, D. & Jakobs, S. Live-cell STED nanoscopy of mitochondrial cristae. *Scientific Reports 2019 9:1* **9**, 1–6 (2019).
7. Chen, F., Tillberg, P. W. & Boyden, E. S. Expansion microscopy. *Science* **347**, 543–548 (2015).
8. Tillberg, P. W. *et al.* Protein-retention expansion microscopy of cells and tissues labeled using standard fluorescent proteins and antibodies. *Nature Biotechnology 2016 34:9* **34**, 987–992 (2016).
9. Sarkar, D. *et al.* Revealing nanostructures in brain tissue via protein decrowding by iterative expansion microscopy. *Nature Biomedical Engineering 2022 6:9* **6**, 1057–1073 (2022).
10. Tanca, A. *et al.* Comparability of differential proteomics data generated from paired archival fresh-frozen and formalin-fixed samples by GeLC-MS/MS and spectral counting. *J. Proteomics* **77**, 561–576 (2012).
11. Tanca, A. *et al.* Critical comparison of sample preparation strategies for shotgun proteomic analysis of formalin-fixed, paraffin-embedded samples: Insights from liver tissue. *Clin. Proteomics* **11**, (2014).
12. Shen, K., Sun, J., Cao, X., Zhou, D. & Li, J. Comparison of different buffers for protein extraction from formalin-fixed and paraffin-embedded tissue specimens. *PLoS One* **10**, (2015).
13. Buzsáki, G. & Mizuseki, K. The log-dynamic brain: how skewed distributions affect network operations. *Nature Reviews Neuroscience 2014 15:4* **15**, 264–278 (2014).
14. Wang, S. S. H. *et al.* Functional Trade-Offs in White Matter Axonal Scaling. *Journal of Neuroscience* **28**, 4047–4056 (2008).
15. Claiborne, B. J., Amaral, D. G. & Cowan, W. M. A light and electron microscopic analysis of the mossy fibers of the rat dentate gyrus. *J. Comp. Neurol.* **246**, 435–458 (1986).
16. Innocenti, G. M., Vercelli, A. & Caminiti, R. The Diameter of Cortical Axons Depends Both on the Area of Origin and Target. *Cereb. Cortex* **24**, 2178–2188 (2014).

17. M'Saad, O. *et al.* All-optical visualization of specific molecules in the ultrastructural context of brain tissue. *bioRxiv* 2022.04.04.486901 (2022).

18. Gunning, B. E. THE FINE STRUCTURE OF CHLOROPLAST STROMA FOLLOWING ALDEHYDE OSMIUM-TETROXIDE FIXATION. *J. Cell Biol.* **24**, 79–93 (1965).

19. Schauflinger, M., Bergner, T., Neusser, G., Kranz, C. & Read, C. Potassium permanganate is an excellent alternative to osmium tetroxide in freeze-substitution. *Histochem. Cell Biol.* **157**, 481 (2022).

20. Dunn, A. K., Smithpeter, C., Welch, A. J. & Richards-Kortum, R. Sources of contrast in confocal reflectance imaging. *Appl. Opt.* **35**, 3441–3446 (1996).

21. Wang, U.-T. T. *et al.* Protein and lipid expansion microscopy with trypsin and tyramide signal amplification for 3D imaging. *Sci. Rep.* **13**, 21922 (2023).

22. Cui, Y. *et al.* Expansion microscopy using a single anchor molecule for high-yield multiplexed imaging of proteins and RNAs. *PLoS One* **18**, e0291506 (2023).

23. Damstra, H. G. J. *et al.* Visualizing cellular and tissue ultrastructure using Ten-fold Robust Expansion Microscopy (TREx). *Elife* **11**, (2022).

24. Yu, C. C. *et al.* Expansion microscopy of c. Elegans. *Elife* **9**, 1–78 (2020).

25. Chen, F. *et al.* Nanoscale imaging of RNA with expansion microscopy. *Nat. Methods* **13**, 679–684 (2016).

26. Chang, J. B. *et al.* Iterative expansion microscopy. *Nature Methods 2017 14:6* **14**, 593–599 (2017).

27. White, B. M., Conwell, A. N., Wu, K. & Baskin, J. M. Lipid Expansion Microscopy. (2022) doi:10.26434/chemrxiv-2022-n4cq4.

28. Wen, G. *et al.* Evaluation of Direct Grafting Strategies *via* Trivalent Anchoring for Enabling Lipid Membrane and Cytoskeleton Staining in Expansion Microscopy. *ACS Nano* **14**, 7860–7867 (2020).

29. Götz, R. *et al.* Nanoscale imaging of bacterial infections by sphingolipid expansion microscopy. *Nat. Commun.* **11**, 6173 (2020).

30. Bolte, S. & Cordelières, F. P. A guided tour into subcellular colocalization analysis in light microscopy. *J. Microsc.* **224**, 213–232 (2006).

31. Lunde, A. & Glover, J. C. A versatile toolbox for semi-automatic cell-by-cell object-based colocalization analysis. *Scientific Reports 2020 10:1* **10**, 1–26 (2020).

32. Otsu, N. THRESHOLD SELECTION METHOD FROM GRAY-LEVEL HISTOGRAMS. *IEEE Trans. Syst. Man Cybern.* **SMC-9**, 62–66 (1979).

33. Valdes, P. A. *et al.* Improved immunostaining of nanostructures and cells in human brain specimens through expansion-mediated protein decrowding. *Sci. Transl. Med.* **16**, eabo0049 (2024).
