# Supplementary material for: Dense, continuous membrane labeling and expansion microscopy visualization of ultrastructure in tissues
Source: Nat Commun. 2025 Feb 12;16:1579. doi: 10.1038/s41467-025-56641-z (PMC11821914; doi:10.1038/s41467-025-56641-z)
Supplement: Supplementary file 2 — Reporting Summary [file 41467_2025_56641_MOESM2_ESM.pdf]

nature portfolio

Corresponding author(s): Ed Boyden

Last updated by author(s): Nov 7, 2024

Reporting Summary

Nature Portfolio wishes to improve the reproducibility of the work that we publish. This form provides structure for consistency and transparency in reporting. For further information on Nature Portfolio policies, see our Editorial Policies and the Editorial Policy Checklist.

Statistics

For all statistical analyses, confirm that the following items are present in the figure legend, table legend, main text, or Methods section.

n/a	Confirmed	
☐	☒	The exact sample size (*n*) for each experimental group/condition, given as a discrete number and unit of measurement
☐	☒	A statement on whether measurements were taken from distinct samples or whether the same sample was measured repeatedly
☒	☐	The statistical test(s) used AND whether they are one- or two-sided<br>*Only common tests should be described solely by name; describe more complex techniques in the Methods section.*
☒	☐	A description of all covariates tested
☒	☐	A description of any assumptions or corrections, such as tests of normality and adjustment for multiple comparisons
☐	☒	A full description of the statistical parameters including central tendency (e.g. means) or other basic estimates (e.g. regression coefficient) AND variation (e.g. standard deviation) or associated estimates of uncertainty (e.g. confidence intervals)
☒	☐	For null hypothesis testing, the test statistic (e.g. *F*, *t*, *r*) with confidence intervals, effect sizes, degrees of freedom and *P* value noted<br>*Give P values as exact values whenever suitable.*
☒	☐	For Bayesian analysis, information on the choice of priors and Markov chain Monte Carlo settings
☒	☐	For hierarchical and complex designs, identification of the appropriate level for tests and full reporting of outcomes
☒	☐	Estimates of effect sizes (e.g. Cohen's *d*, Pearson's *r*), indicating how they were calculated

Our web collection on statistics for biologists contains articles on many of the points above.

Software and code

Policy information about availability of computer code

Data collection	Spinning disk confocal microscope with NIS element software and electronmicroscope.
Data analysis	MATLAB, R, Python, Fiji/ImageJ, MS excel, and https://github.com/TAYmit/umExM

For manuscripts utilizing custom algorithms or software that are central to the research but not yet described in published literature, software must be made available to editors and reviewers. We strongly encourage code deposition in a community repository (e.g. GitHub). See the Nature Portfolio guidelines for submitting code & software for further information.

Data

Policy information about availability of data

All manuscripts must include a data availability statement. This statement should provide the following information, where applicable:

- Accession codes, unique identifiers, or web links for publicly available datasets
- A description of any restrictions on data availability
- For clinical datasets or third party data, please ensure that the statement adheres to our policy

Data is available at https://osf.io/qtbek/

Research involving human participants, their data, or biological material

Policy information about studies with human participants or human data. See also policy information about sex, gender (identity/presentation), and sexual orientation and race, ethnicity and racism.

Reporting on sex and gender	N/A
Reporting on race, ethnicity, or other socially relevant groupings	N/A
Population characteristics	N/A
Recruitment	N/A
Ethics oversight	N/A

Note that full information on the approval of the study protocol must also be provided in the manuscript.

Field-specific reporting

Please select the one below that is the best fit for your research. If you are not sure, read the appropriate sections before making your selection.

☒ Life sciences ☐ Behavioural & social sciences ☐ Ecological, evolutionary & environmental sciences

For a reference copy of the document with all sections, see nature.com/documents/nr-reporting-summary-flat.pdf

Life sciences study design

All studies must disclose on these points even when the disclosure is negative.

Sample size	We attempted to replicate the experiments at least 2-3 times. The number of replications for each experiment is clearly described in the manuscript.
Data exclusions	None
Replication	All the replications for this study were successful.
Randomization	Randomization was not applicable to this study, as our aim was solely to showcase our technology using standard cell lines and mice.
Blinding	Blinding was not applicable to this study, as our focus was solely on demonstrating our technology using standard cell lines and mice.

Reporting for specific materials, systems and methods

We require information from authors about some types of materials, experimental systems and methods used in many studies. Here, indicate whether each material, system or method listed is relevant to your study. If you are not sure if a list item applies to your research, read the appropriate section before selecting a response.

Materials & experimental systems

n/a	Involved in the study	
☐	☒	Antibodies
☐	☒	Eukaryotic cell lines
☒	☐	Palaeontology and archaeology
☐	☒	Animals and other organisms
☒	☐	Clinical data
☒	☐	Dual use research of concern
☒	☐	Plants

Methods

n/a	Involved in the study	
☒	☐	ChIP-seq
☒	☐	Flow cytometry
☒	☐	MRI-based neuroimaging

Antibodies

Antibodies used	anti-TOM20 (rabbit, Cell Signaling Tech., catalog no.42406S)<br>anti-TOM20 (mouse, Santa Cruz Biotech., catalog no.sc-17764)<br>anti-NUP98 (rabbit, Cell Signaling Tech., catalog no.2597S)<br>anti-MBP (rabbit, Cell Signaling Tech., catalog no.78896S)<br>anti-MBP (rabbit, Abcam, catalog no.ab40390)

	anti-MBP (chicken, AVES, catalog no.AB_2313550)<br><br>anti-GFP (nanobody; ChromoTek, catalog no. gba647n)<br>anti-GFP (nanobody; ChromoTek, catalog no. gba488)<br>anti-SV2A (rabbit, Abcam, catalog no. ab32942)<br>anti-PSD95 (rabbit, Thermo Fisher, catalog no. MA1-046)<br>anti-Rabbit ATTO 647N (Rockland Immunochemicals, catalog no. 50-194-3924
Validation	All the antibodies used in this study are commercially available. The validity of each primary antibody was confirmed using information provided on the vendor's website.

Eukaryotic cell lines

Policy information about cell lines and Sex and Gender in Research

Cell line source(s)	HEK 293 (ThermoFisher) HeLa (ATCC) U2OS(ATCC)
Authentication	HEK 293 cells can be obtained from ThermoFisher, while HeLa and U2OS cell lines are available from ATCC.
Mycoplasma contamination	Not tested for mycoplasma contamination.
Commonly misidentified lines<br>(See ICLAC register)	We did not use any cell lines that are commonly misidentified in this study.

Animals and other research organisms

Policy information about studies involving animals; ARRIVE guidelines recommended for reporting animal research, and Sex and Gender in Research

Laboratory animals	Wild type C57BL/6 mice from Taconic. Thy1-YFP mice from JAX.
Wild animals	N/A
Reporting on sex	Used without regard to sex.
Field-collected samples	N/A
Ethics oversight	All procedures involving mice (Thy1-YFP-H, 6–8 weeks of age from JAX, used without regard to sex) were performed in accordance with the US National Institutes of Health Guide for the Care and Use of Laboratory Animals and approved by the MIT Committee on Animal Care.

Note that full information on the approval of the study protocol must also be provided in the manuscript.

Plants

Seed stocks	N/A
Novel plant genotypes	N/A
Authentication	N/A
