# Supplementary material for: Dense, continuous membrane labeling and expansion microscopy visualization of ultrastructure in tissues
Source: Nat Commun. 2025 Feb 12;16:1579. doi: 10.1038/s41467-025-56641-z (PMC11821914; doi:10.1038/s41467-025-56641-z)
Supplement: Supplementary file 3 — Description of Additional Supplementary Files [file 41467_2025_56641_MOESM3_ESM.pdf]

Supplementary Movies

Supplementary Movie 1

Stack of confocal images of expanded mouse brain tissue (somatosensory cortex) after umExM processing, showing pGk13a staining of the membrane.

Supplementary Movie 2

Stack of confocal images of expanded mouse brain tissue (hippocampus, CA2) after umExM processing, showing pGk13a staining of the membrane.

Supplementary Movie 3

Stack of confocal images of expanded mouse brain tissue (hippocampus, dentate gyrus) after umExM processing, showing pGk13a staining of the membrane.

Supplementary Movie 4

Stack of confocal images of expanded mouse brain tissue (corpus callosum) after umExM with double gelation processing, showing pGk13a staining of the membrane.

Supplementary Movie 5

Stack of confocal images of expanded mouse brain tissue (corpus callosum) after umExM with double gelation processing, showing pGk13a staining of the membrane.

Supplementary Movie 6

Stack of confocal images of expanded mouse brain tissue (somatosensory cortex) after the iterative form of umExM processing, showing pGk13a staining of the membrane.
